# Late Pleistocene to early Holocene high-quality quartz crystal procurement from the Valiente quarry workshop site (32°S, Chile, South America)

**DOI:** 10.1371/journal.pone.0208062

**Published:** 2018-11-29

**Authors:** César Méndez, Amalia Nuevo Delaunay, Roxana Seguel, Antonio Maldonado, Ismael Murillo, Douglas Jackson, Eugenio Aspillaga, Roberto Izaurieta, Víctor Méndez, Macarena Fernández

**Affiliations:** 1 Centro de Investigación en Ecosistemas de la Patagonia (CIEP), Coyhaique, Aisén, Chile; 2 Centro Nacional de Conservación y Restauración, Servicio Nacional del Patrimonio Cultural, Santiago, Región Metropolitana, Chile; 3 Centro de Estudios Avanzados en Zonas Áridas, Universidad de La Serena, La Serena, Coquimbo, Chile; 4 Servicio Nacional de Geología y Minería, Providencia, Santiago, Región Metropolitana, Chile; 5 Sociedad Malacológica de Chile, Santiago, Región Metropolitana, Chile; 6 Departamento de Antropología, Universidad de Chile, Santiago, Región Metropolitana, Chile; 7 Independent researcher, Santiago, Región Metropolitana, Chile; 8 Universidad Alberto Hurtado, Santiago, Región Metropolitana, Chile; New York State Museum, UNITED STATES

## Abstract

The procurement of high-quality lithic resources is amongst the most indicative processes of decision-making in the archaeology of early human groups peopling the Americas. Directly dated deposits from quarry workshops have been absent of the late Pleistocene record of South America. We present the results of the excavations of a high-quality translucent quartz crystal workshop that yielded radiocarbon-dated coherently layered stratigraphic deposits that shed light into the behavior of the initial stages of lithic procurement. Based on a detailed analysis of the context of the Valiente site (32° S, Chile, South America), we discuss the stages of bifacial production of point technology. The deposit produced evidence of cumulative occupations over the period between 12,630 and 11,320 calibrated years before present. This ~1,300-year span is coincidental with a major environmental step-wise drying trend as indicated by the local and regional pollen records. Furthermore, it is synchronous to the process in which natural landscapes became the earliest taskscapes in the region, thereby encompassing major cultural changes related to the organization of the land use. These results are discussed in the frame of contemporaneous archaeological data to discuss specific aspects of technology and decision-making of the earliest settlers of South America.

## Introduction

Though crucial as a stage in lithic procurement, and therefore mandatory for understanding technological behaviors arising from it, quarrying of raw toolstones is an elusive topic for the early peopling of South America. Lithic quarry workshops possess singular qualities because they constitute fixed points in space where above-average quality toolstones are localized and therefore have profound implications for the organization of economy [1–4]. This often results in high occupational redundancy because such locations were important for planning procurement activities in space and time [5]. Though immensely significant for identifying a strong human signature in the landscape, high intra-site redundancy may in turn lead to superimposed occupations, frequently resulting in palimpsests, even spanning several millennia [6–9]. Inherent difficulties for the interpretation of such sites, for example the need of considering deposits as averaging multiple occupations can be problematic, especially for understanding variation in their use [10, 11]. Careful excavating techniques, direct AMS dating, and above all, a detailed knowledge of site formation processes, are required for the proper interpretation of such sites. Only through a comprehensive understanding of the characteristics of the archaeological record of quarry workshops at a site scale can we start to disentangle the initial stages in technological decision-making of the earliest inhabitants of the Americas.

Despite the fact that quarry workshop sites are known for late Pleistocene North America [12–14], these are yet poorly documented for the earliest inhabitants of South America, especially in arid environments where sedimentation is often low and stratified and well-dated deposits with such evidence are rare [15, 16]. There is abundant geochemical provenance information and typological examples indicating that quarries and zones rich in raw materials were being used during the Pleistocene-Holocene transition in South America [8, 17–22]. Also, there is available data concerning early toolstone transport across large spatial scales [23–25]. However, detailed excavations on sites where high-quality toolstones occur naturally are few, and they are non-existent when it comes to ages of the Pleistocene-Holocene transition. Documenting such a record not only fills the void of characterizing the initial stages in lithic procurement but is altogether crucial for understanding the evolution of technological behaviors in the landscapes of the earliest Americans.

**Fig 1:**
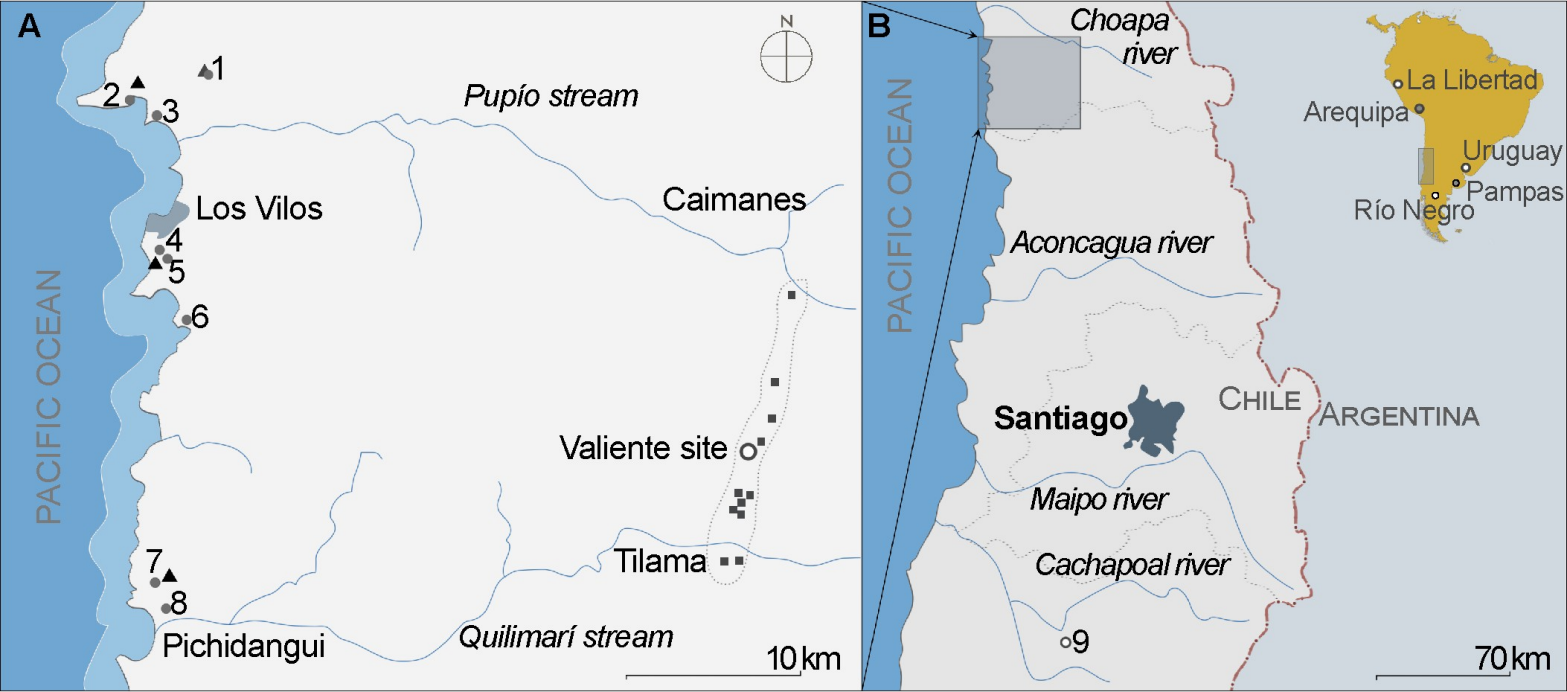
Map of the study area showing sites and regions discussed. A. Map at ~32° S, B. Central Chile, C. South America showing: archaeological sites (gray circles), archaeological sites with fishtail points in quartz (white circles), locations sampled for quartz natural distribution (squares), paleoenvironmental coring sites (triangles) discussed in this study. 1. Quebrada Santa Julia, 2. Punta Ñagué, 3. Punta Penitente, 4. Quereo Norte, 5. Quereo Perfil, 6. Punta Purgatorio. 7. Palo Colorado, 8. Pichidangui, 9. Taguatagua 2.

Currently, ages exceeding 14,600 calibrated years before present (cal BP) are being discussed as the temporal markers for the early peopling of the southern subcontinent [26–29]. However, no context of such age has yielded evidence of quarrying toolstones. In this paper we present evidence for such stage of technological behavior for a region where other sites have previously produced independently-dated contexts of the Pleistocene Holocene transition [30, 31]. The earliest site in the semiarid north of Chile (SAN) is Quebrada Santa Julia, a rapidly-buried 8-to-5-cm sedimentary unit that yielded the evidence of a transitory camp, where a limited set of activities were carried out over a very brief time span [30]. Averaged dates place the occupation at 12,990–12,730 cal BP (2σ), a time when the sea margin was ~8 km distant from the site [32–34]. Roughly 40% of the lithic debris corresponds to evidence indicative of knapping high-quality translucent quartz crystal for the production of bifacial (N = 2) and marginally retouched tools (N = 2) [32]. Not only this site showed that quartz was significant among earliest settlers in the region, but scattered lithic materials, especially projectile points at various late Pleistocene-to-early Holocene sites in the coast around 32° S, also underscored its regional importance (Fig 1) [35]. Quartz is a non-local toolstone in this zone and crystal quartz of such properties is not ubiquitous. According to available geological maps, quartz occurs in abundance along a longitudinal strip 35 km in average from the coastline [36]. Guided by its geological occurrence, we engaged on a systematic surface survey searching for early evidence of its exploitation. Quartz was a primary lithic resource represented in archaeological assemblages along this transect, though high-quality crystal quartz use was just limited to one site.

The Valiente site is a lithic workshop site located within a modern quartz quarry with occasional small-scale operations. Bifacial fragments and bifacial thinning flakes observed on an exposed profile, indicated a stratified archaeological deposit with outstanding quality toolstone, which bears remarkable similarities to the one excavated in the Quebrada Santa Julia site [37]. Secure late Pleistocene dates, a constant depositional rate and spatial associations of material remains allow discussing technological decisions of the initial stages of lithic procurement for the earliest settlers of the region. This paper addresses the site context, geoarchaeology and material assemblages of a quartz workshop site spanning a period in which the SAN broadly underwent a transformation into a semiarid environment. We discuss the implications of a finding such as Valiente site in a supraregional scale regarding two significant issues: a chronologically well-constrained quarry workshop site and its implications regarding mobility and use of space in settings with a high-quality toolstone concentration.

## Study area and paleoenvironment

The Valiente site (32°01’42’S; 71°09’39”W; 714 masl) is located 6 kilometers north of the town of Tilama in the southernmost limit of the SAN (Fig 1). The SAN (~26° to 32° S) is an environmental band characterized by dry summers and infrequent winter precipitation as a product of the seasonal latitudinal migration of the northern limit of the Westerlies Wind Belt in its interaction with the high-pressure cell of the Subtropical Anticyclone of the South Pacific [38]. The climate of the study area is Mediterranean Xeric-Ocean (BSks), with an average precipitation of 231 mm/year and an average temperature between 19.3–9.4°C [39, 40]. Interannual and multi-decadal climate fluctuation in this area is mainly controlled by El Niño Southern Oscillation (ENSO), with warmer oceanic phases expressed as years with higher and occasionally torrential rainfall (El Niño), and dryer years where precipitation concentrates in winter (La Niña) [38, 41].

The southernmost part of the SAN (31° to 32° S) is the narrowest segment of Chile. Here the coastal margin is separated from the highest Andean divide by ca. 100 km. The Valiente archaeological site is located 34 kilometers from the coast, within a 5.9 km^2^ intermountain enclosed basin where the El Naranjo ravine, one of the two tributaries of Qulimarí river, origins [42]. Vegetation is sclerophyll shrubland [40]. In this area, topographic characteristics often preclude the introduction of air-masses carrying moisture from the Pacific Ocean thus making it particularly arid [43]. Available faunal taxa are limited to foxes (*Lycalopex*) and various rodent species [44], though mammalian richness during the Pleistocene-Holocene transition should have been higher as suggested by regional archaeological and paleontological records [45–47].

Available regional paleoenvironmental records spanning the Pleistocene-Holocene transition have been either obtained in the high mountains or near the coastal margin. The terminal Pleistocene regional climate was wetter than today as documented by local coastal archives and other terrestrial and marine sites in the region [48, 49]. The pollen record obtained from stratigraphic deposits overlaying the human occupation at the Quebrada Santa Julia site suggests climate started drying after 11,200 cal BP, though wetland expansions inferred at 10,500 cal BP and at 9,200 cal BP are indicative of increased regional moisture, thus implying that the shift towards drier conditions was not unidirectional [50]. This step-wise drying trend is consistent with other regional offshore sedimentary records [51, 52]. At 8,600 cal BP the Palo Colorado coastal sediment core shows evidence suggestive of a major and widespread regional aridity [53].

## Materials and methods

**Fig 2:**
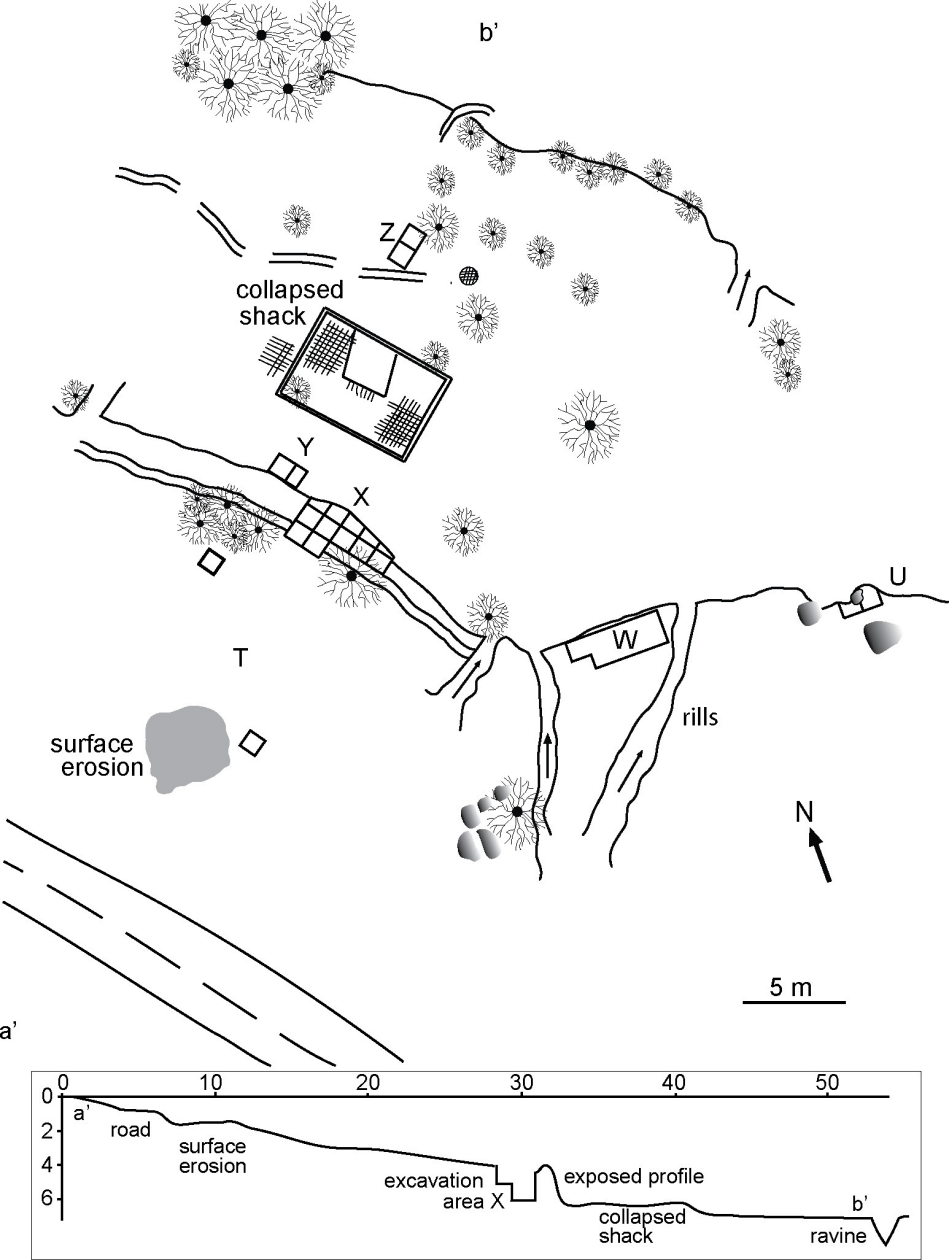
General plan and schematic cross section of Valiente site. Capital letters stand for the designated areas in the site.

Four field seasons between 2009 and 2012 comprise the total time/effort devoted to our work in Valiente. Field research permit #2707/11 was granted by the Consejo de Monumentos Nacionales, Chile. A total of ~18 m^2^ were excavated in 6 different sectors (labeled: X-Z) in the site (Fig 2). Most excavations were intended for defining the stratigraphic extension of lithic material, which we currently know is almost exclusively recorded in area X and in the adjacent slope labeled as area T and discontinuously and to a lesser degree in area U. Excavations focused mainly on area X (~9 m^2^). Given that no layering or stratigraphic changes were visible, the excavated sediments were subdivided in artificial 10-cm levels. To compensate for this, we recorded tridimensional measures for all bone fragments, charcoal speckles and artifacts >2 cm, resulting in the piece-plotting of 4,162 individual specimens. Recovery methods also included the careful excavation of features and sampling for flotation and sediment analysis. All sediments were dry-sieved in 2 mm meshes. The total amount of material recovered totalizes 14,416 specimens (95.82% complete lithics and fragments, 3.72% bone fragments, and 0.46% charcoal speckles).

Eleven AMS radiocarbon (^14^C) dates were used to establish the chronology of human occupations and to characterize the sedimentation rate [54]. All dates in this paper were calibrated into years before present (cal BP) with OxCal 4.3 using the ShCal13 curve and are expressed in 2σ ranges [55, 56]. To stablish a minimum number of occupational events at the site level, two or more dates were combined whenever they were statistically indistinguishable at α = 0.05 [57]. These were used for discussing the time span of occupation of this locality.

Lithic material was analyzed using technological criteria that focused on assessing the completeness, tool and debitage classes and specific descriptive technological attributes [32, 58]. In order not to overestimate the frequency of knapping activities, lithic quantifications distinguished a minimum number of elements considering all complete pieces and those bearing striking platforms [59]. The total mass of each specimen was recorded to compare the overall raw material processed at the site. Varieties of quartz were recorded at a macroscopic level and grouped by translucency in order to assess fracture quality [58]. To characterize the lithic taphonomy of the studied assemblage we recorded traces left in the surfaces of artifacts by alteration processes on a subsample of elements with tridimensional referencing from unit B2 (N = 307). These were compared to alteration patterns resulting from controlled observations of surface specimens from the slope on area T (N = 59) and with patterns described elsewhere [60–62] to identify the taphonomic agents that would have contributed to the formation of the deposit.

A preliminary assessment of faunal remains underscored the overall state of fragmentation, burning and limited taxonomically diagnostic value of a bone sample from the first excavated units [45]. Consequently, anatomical and taxonomical definitions were based on few specimens compared to reference collections (Anthropology Department, Universidad de Chile), and hence, they need to be considered with caution. Most taxonomic identifications were limited to the class level. Considering the above, remains were only quantified using the Number of Identified Specimens (NISP) per taxa [63–65]. Given the dominance of traces from fire exposure, their occurrence and extension were recorded [66]. To determine other potential sources for color, six bone samples from different excavation levels were analyzed using a TESCAN (VEGA3) scanning electron microscope (SEM) with a Bruker Quantax energy dispersive spectrometer (EDS) at the laboratory for Electronic Microscopy of Universidad de Santiago (Chile).

All specimens in this study (i.e., lithic, bone and charcoal fragments) are curated in the Anthropology Department, Universidad de Chile (address Capitán Ignacio Carrera Pinto 1045, Ñuñoa Santiago, 7800284). They are available prior consultation to the collection curator (http://www.facso.uchile.cl/antropologia/patrimonio/55923/colecciones-de-antropologia). All specimens discussed in the manuscript are referred to a specimen number in the manuscript and figures.

## The Valiente quarry workshop site

Quartz occurs naturally over a well-defined north-south stripe, distant 34–37 km from the coast, extending between 31°37’S—31°56’S, and discontinuously between 30°56’S—32°10’S [36]. It corresponds to intrusive pegmatitic bodies within granites of the Illapel Superunit of the Superior Cretacic, where white non-translucent large masses of quartz dominate, alongside occasional highly translucent fragmented quartz crystals [36, 67]. This mineral source is of industrial importance as judged by recent productive yields [68], as well as the direct observation of ongoing mining in the environs.

**Fig 3:**
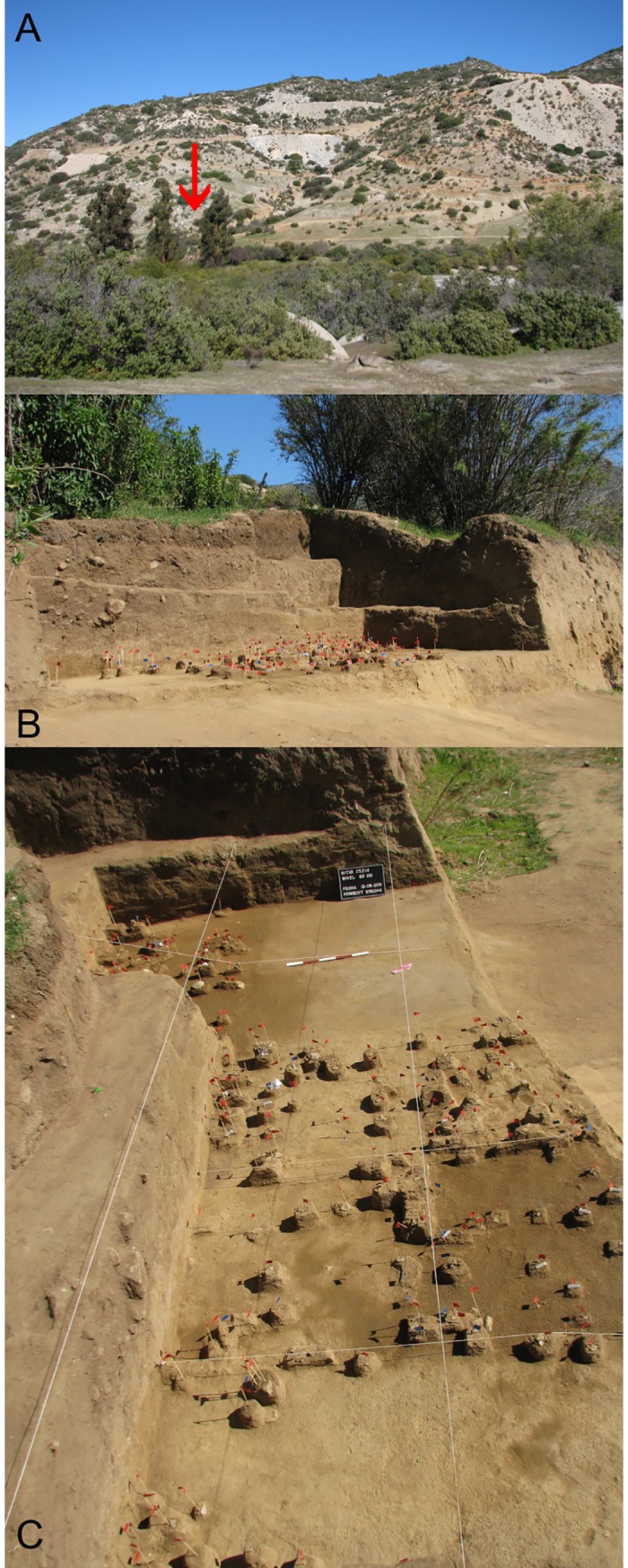
Images of Valiente site. A. General view of El Naranjo ravine in the front and the quartz mine in the back; the red arrow points to excavation area X. B. and C. section and plan photographs of the excavation at 62 cm (12,730–12,590 cal BP) of area X. Red flags: lithics, blue flags: bone and charcoal fragments.

Given its spatial proximity to the earliest human occupation recorded at Quebrada Santa Julia, quartz occurrence and prehistoric use were assessed through surface surveys on a 1-km wide transect along the 15 km separating the towns of Caimanes and Tilama [37]. Availability and quality of potential toolstone sources were evaluated and the archaeological surface record was sampled [69]. Eleven points show mainly white non-translucent quartz and only two of them yielded translucent crystal [32]. This toolstone was observed mainly as concentrated surface outcrops and rarely as stratified veins. Most occurrences exhibit fracture qualities incompatible with bifacial flaking, which is a common feature with quartz [70]. Along this area, twenty-four archaeological sites were recorded, among them only four without pot sherds (last 2000 years in age) and or sufficient size/artifact densities as to conduct studies. Tests excavations were conducted at three sites (L.V.232/D8.2; CT21) of which only the Valiente site (CT14) combined high-quality translucent quartz bifacial artifacts and chipping debris in stratified deposits over a well-defined 7-m exposed profile (Fig 3A) [35, 37, 71]. Valiente site extends over an approximate area of 590 m^2^ where we concentrated field work (S1 Fig).

### Geomorphology, stratigraphy and radiocarbon chronology

**Fig 4:**
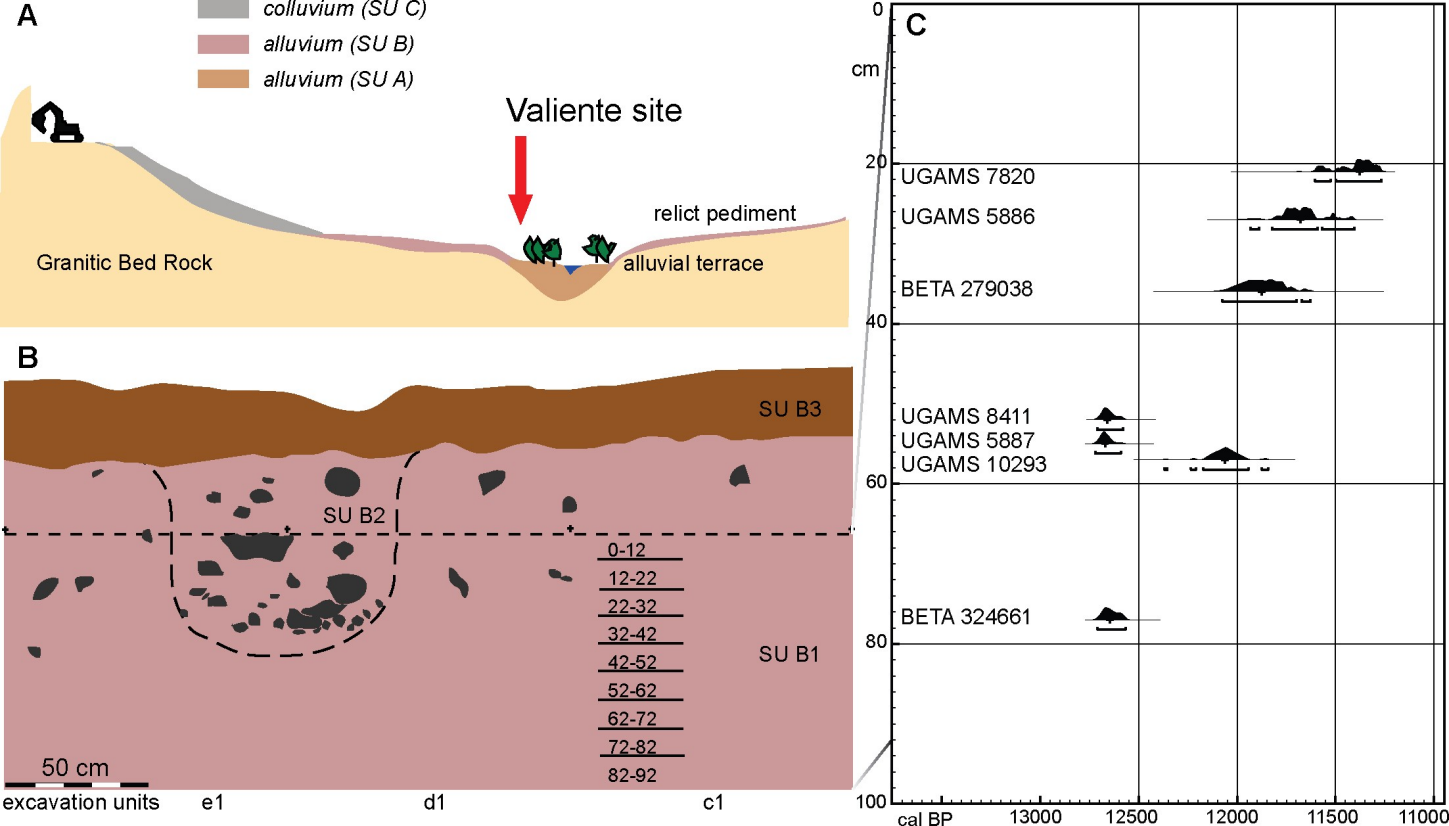
Stratigraphy of Valiente site. A. schematic site profile showing stratigraphic units, B. southwest stratigraphy of excavated units E1, D1 and C1 from area X showing stratigraphic sub units and excavated levels, C. depth/age model of the archaeological deposit.

The local geomorphology shows an alluvial terrace demarcated at the base by the rocky substrate, whose cover interfingers sideways with the alluvial deposits that overlie a remnant pediment on the slopes of the basin (Fig 4A). Pediments are a typical landform of arid and semiarid environments, where gentle slopes (<5%) with flat to slightly concave surfaces promote alluvial movement in a parallel manner rather than producing channel incisions [72].

Three basic units comprise the stratigraphic sequence at Valiente; aggrading alluvial deposits (stratigraphic unit (SU) A), slope deposits (SU B) and the colluvium (SU C) enhanced by recent small-scale quartz mining SU A, are alluvial deposits including sands and silts, with poorly-sorted irregular pebble to gravel matrix-supported particles. Clast composition varies from polymictic-andesitic to quartz fragments and predominant feldspar. This SU presents erosive incisions of up to 10 meters deep and occasional terrace levels, some with incipient soil formation. Two radiocarbon dates, one on organic matter and the other on charcoal from this unit, resulted in modern ages. SU B are slope deposits composed by mainly quartz and feldspar sands, silts and clays with matrix supported quartz angular fragments and other lesser represented lithologies. However, this SU presents occasional rills of 0.2 to 4 m wide and up to 4 m deep, which are seldom filled with matrix supported deposits conformed by gravel size quartz fragments included in a sand and clay sized matrix (Fig 4B). These features indicate localized erosions, possibly associated to heavy rainfall episodes. The SU B varies in thickness from 20 cm in the uppermost part of the slope to up to 5 m in its contact with SU A. A detailed stratigraphy of the excavated area X is available in S1 Appendix.

Eleven radiocarbon dates have been used to constrain the chronology of SU B and the anthropogenic deposition at the Valiente site. Nine age controls are from area X (Table 1). Plotted against depth, seven of them indicate a constant sedimentation rate throughout ~60 of the ~90-cm depositional package with human evidence (Fig 4C). The upper section (first 10 to 20 cm) has proven to be affected by an artificial channel especially in the southwestern excavation units (A3 and B3), as shown by incipient soil formation (Fig 4B). It caused the incorporation of recent organic material (UGAMS 7819) and vertical displacement of some samples (UGAMS 10295). This is the case of a date on a Mylodontidae dermal bone (#2788), which besides being anomalously recent, is regarded as relocated given that it was recovered beneath the water channel, an area more prone to liquefying sediments. Regarding the lower section (beneath ~50 cm of depth), three (UGAMS 8411, UGAMS 5887, BETA 324661) out of four dates are statistically indistinguishable and can be pooled into an average age of 12,690–12,550 cal BP (10,686 ±19 BP), which can be regarded as the initial time for the anthropogenic deposition at Valiente. After recognizing and isolating potential disturbances, we can observe an orderly deposited sedimentary sequence spanning roughly 1,300 years across the Pleistocene/Holocene transition.

**Table 1:** Radiocarbon chronology of the Valiente site.

Spatial reference	Depth	Lab. code	Age (sd) yr BP	δ^13^C	2σ cal BP	Material
**Area X, Unit D2**	10 cm	UGAMS 7819	130 ±25	-23.9	260–0	Charcoal^a^
**Area X, Unit C1**	21 cm	UGAMS 7820	9,970 ±30	-20.5	11,600–11,230	Charcoal
**Area X, Unit B1**	22–32 cm	UGAMS 5886	10,090 ±30	-20.9	11,760–11,350	Charcoal
**Area X, Unit A2**	36 cm	BETA 279038	10,180 ±50	-19.5	12,010–11,400	Charcoal
**Area X, Unit C2**	52 cm	UGAMS 8411	10,680 ±30	-24.8	12,690–12,550	Charcoal
**Area X, Unit A3**	52 cm	UGAMS 10295	8,560 ±40	-22.5	9,550–9,460	Bone^b^, Mylodontidae
**Area X, Unit B1**	55 cm	UGAMS 5887	10,700 ±30	-23.8	12,700–12,550	Charcoal
**Area X, Unit C1**	52–62 cm	UGAMS 10293	10,290 ±30	-24.8	12,070–11,810	Charcoal^b^
**Area X, Unit B2**	72–82 cm	BETA 324661	10,670 ±40	-26.1	12,700–12,440	Charcoal
**Area T, Unit 1**	150–160 cm	UGAMS 10294	10,110 ±30	-25.2	11,810–11,390	Charcoal
**Area U, Unit A1**	30–40 cm	UGAMS 7818	5,110 ±30	-17.7	5,910–5,720	tooth, *Homo sapiens*

All radiocarbon dated material comes from SU B-1. All charcoal samples are isolated speckles.

^a^Associated with the recent anthropogenic channel.

^b^Possible vertical displacement.

### Intrasite distribution

The anthropogenic deposit at Valiente site was discovered through the observation of quartz crystal bifaces and chipping debris exposed in an artificial profile associated to a collapsed shack of recent construction. This sector, labeled X, was the area where main excavations concentrated and, consequently, yielded most of the archaeological remains. These consist almost exclusively of lithics, small charred bone remains and small charcoal isolated particles. They were excavated from SU B1. No hearths or other anthropogenic features were recorded alongside the lithics and organic remains. The lack of visible stratigraphic changes within this SU made it necessary to proceed excavation by 10-cm artificial levels which became the minimal unit of temporal reference. All associations were attained through the piece plotting of individual materials within these levels.

**Fig 5:**
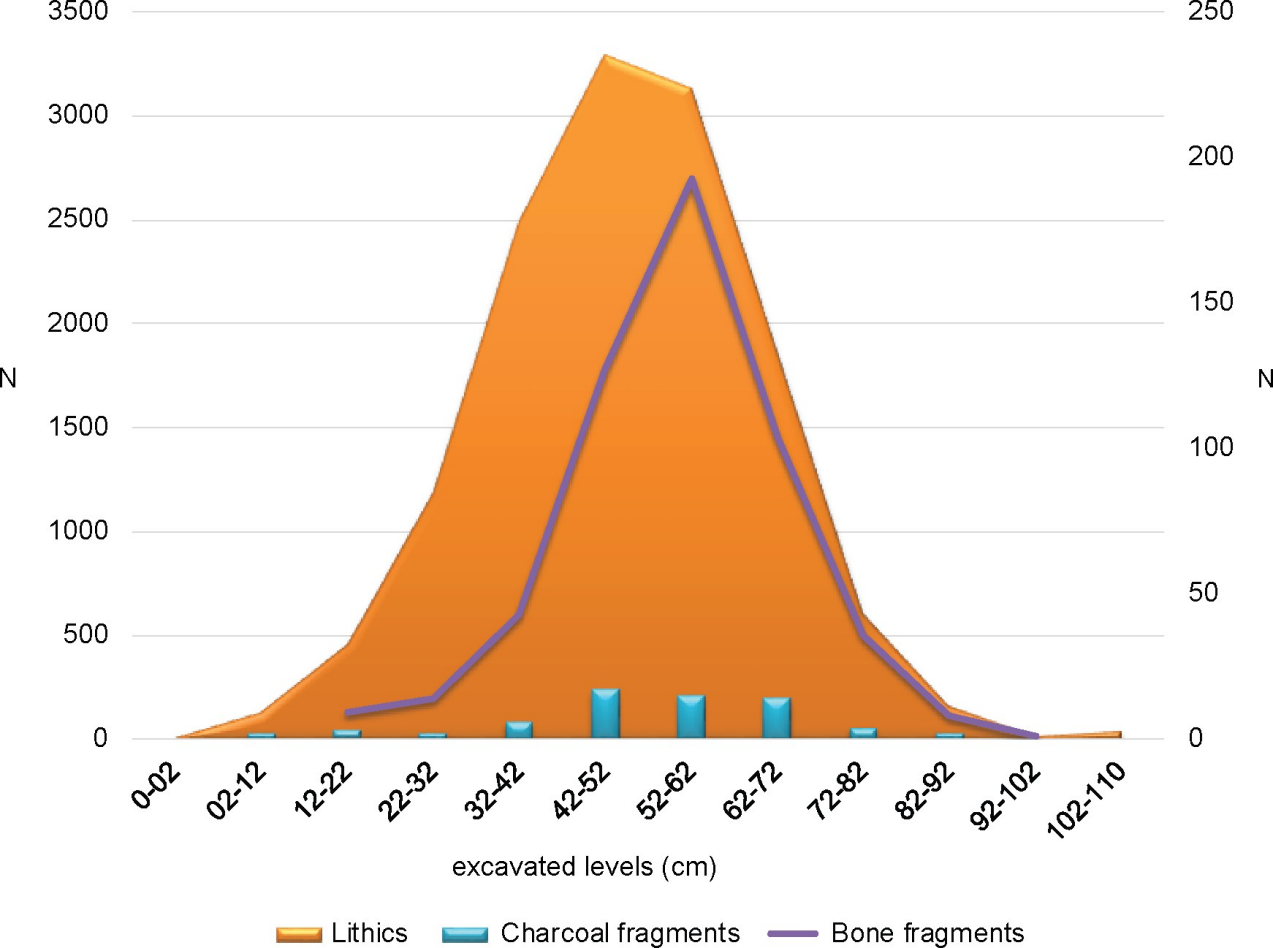
Distribution of archaeological material across excavated levels. Left scale is for lithic specimens (complete and fragments), right scale is for bone and charcoal fragments.

As expected for a slope deposit, all recovered material is likely to have moved from its original place of deposition. This is consistent with the absence of localized features and the overall state of organic material, which was recovered in very low proportion and showing advanced deterioration. However, the fact that radiocarbon dates on speckles of charcoal are in an ordered sequence and that they indicate a constant deposition rate over more than one millennium, suggest that movement along the gentle slope must have occurred over a relatively short distance before final burial. Hence, while material associations are not to be regarded necessarily as primary, nonetheless they should be considered accurate within the time frame they occur. Only the initial 30 cm of excavation units E and D showed a localized completely-filled channel incision with matrix-supported clasts affecting the integrity of the deposit (S1 Appendix). Lithic debris and other materials were minorly represented within this feature, and we interpreted they must have been hauled by alluvial movement along this crevice when it formed. For the rest of SU B1, materials are distributed in a unimodal fashion peaking in levels between 42 and 62 cm (Fig 5). The distribution is similar for lithics and bones and slightly more even across the excavated levels regarding charcoal material. The decreased frequency of specimens below 72 cm in depth suggests their likely vertical displacement in the deposit, something consistent with the statistically indistinguishable dates obtained at these different levels (S2 Fig).

**Fig 6:**
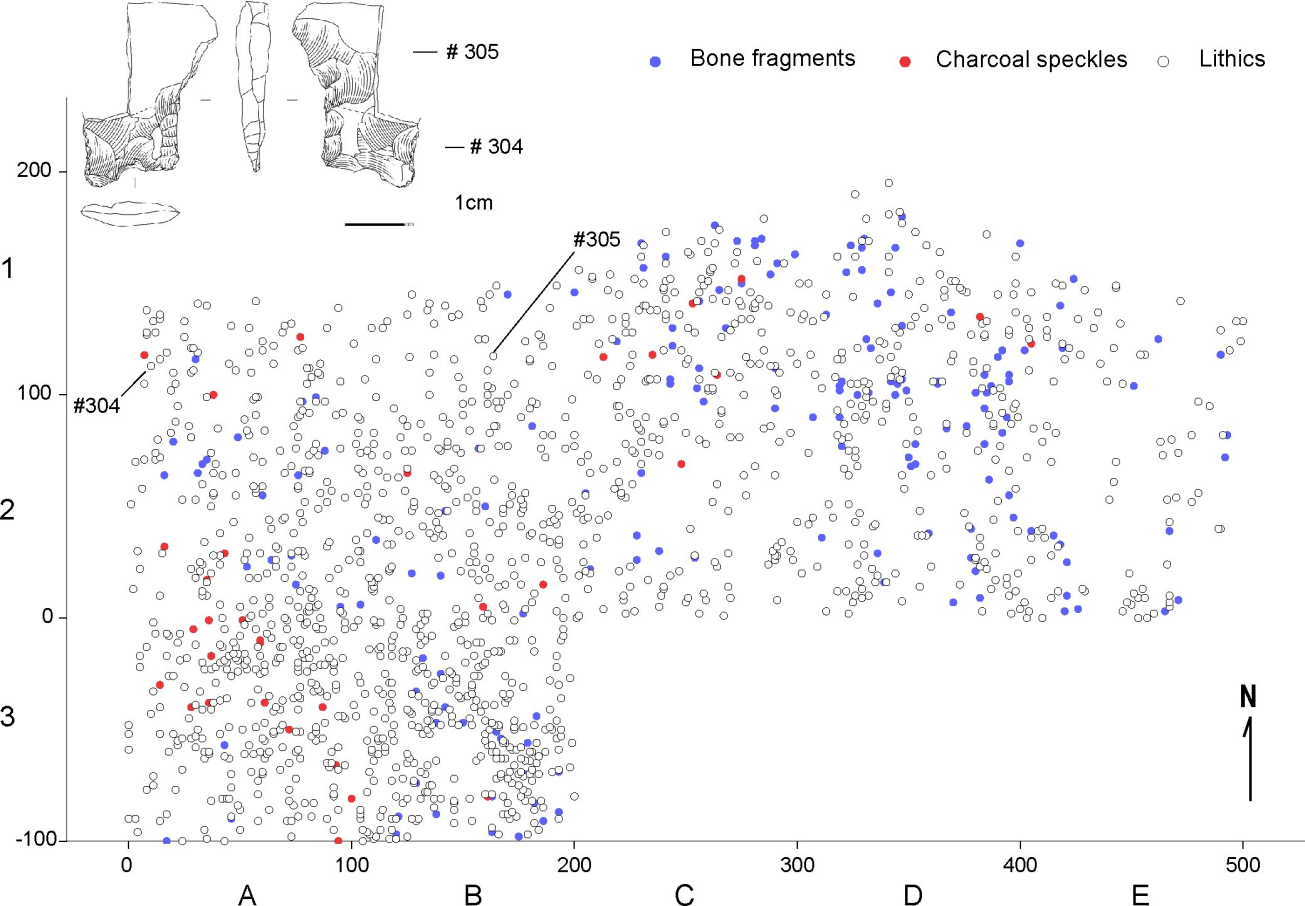
Spatial distribution of archaeological material between 12,690–12,550 cal BP. Considers piece plotted specimens between 52 and 82 cm; X and Y axis are in cm.

A coarse-grained spatial analysis shows lithic material is distributed unevenly across excavated units and levels. Greater frequencies were observed in the upper levels of the southern units, as well as in the lower levels of the northern units (S3 Fig), a distribution which is interpreted as produced by the slope of the site. A finer scale spatial analysis, based on the distribution of 3,938 mapped lithic artifacts, corresponding mostly to debitage, 323 pieces of mapped bone fragments and 67 individually located charcoal speckles, indicates a close association between specimens throughout the excavated deposit (S4 Fig). The type of activities conducted at the site, e.g. the early stages in lithic knapping, plus the overall brittleness of quartz, produced intense fracturing of the specimens (see below), thus limiting a relevant numbers of refits that may illustrate the spatial dimension of activities conducted at the site, as well as other site formation processes [73, 74]. However, one refit between the stem and a medial fragment of the body of a fishtail-type point (#304 and 305) in an early stage of manufacture indicates a close spatial association (Fig 6). This was attained at a level confidently dated between 12,690–12,550 cal BP and further lends support to the idea that despite movement across the slope cannot be ruled out, it must have occurred over very short distances.

On the one hand, test excavations in area Y, immediately to the northwest, and in area Z to the northeast, were designed to assess the vertical distribution of the anthropogenic deposit and whether it continued into the alluvial terrace (SU A). These excavations confirmed that the archaeological material was restricted to the same excavated levels as in X and that no material extended beyond SU B. On the other hand, one of the two 1-m^2^ test excavations in area T, the slope adjacent to the main excavation, demonstrated that the archaeological deposit continues beneath the inclined surface 10 meters to the southwest of area X. A radiocarbon age of 11,810–11,390 cal BP from unit 1 at a depth of 1,5 m was obtained in association to quartz flakes and isolated charcoal speckles, which suggest this is the top of the anthropogenic deposit.

Area U corresponds to a separate archaeological context as suggested by the discontinuity of archaeological material southeast of area X, since chipping debris and other material is absent in exposed profiles, in erosion crevices and in the test excavations at area W (Fig 2). However, bioarchaeological material, consisting of human teeth and one small skull fragment concentrated in less than 625 cm^2^, was visible in a profile of area U. A 2-m^2^ unit, 25 meters from area X, excavated adjacent to this profile revealed a partially preserved human skeleton (S2 Appendix). Though remarkably deteriorated, some bones were still in anatomical position. Material recorded alongside this funerary context consisted of some faunal remains, one grinding stone, and flakes of quartz, basalt and siliceous toolstones; thus, they are markedly different to the assemblages on area X. A direct age of 5,910–5,720 cal BP on an upper first molar of this individual confirmed chronological differences despite being in the same SU as the archeological material in area X.

### Archaeological assemblages of area X

#### Lithic technology

The lithic material dominates the archaeological assemblage at Valiente. It includes 13,813 specimens; mainly debitage pieces of which quartz comprises 99.05% of the sample, value expected to occur near a good quality source. This proportion is evenly distributed across excavated levels (range between 96.59% to 100%). Fragmentation is significantly high (Table 2). Complete and proximal pieces comprise only 20.93% of the assemblage, a proportion that remains even throughout the excavated section (range between 13.16% to 24.43%). With a mean weight of ~1 g per specimen, the sum of knapped quartz in area X totalizes 13.5 kg. Its distribution across excavated levels closely mimics the distribution of lithic counts in Fig 5.

**Table 2:** Summarized frequencies of the lithic assemblage.

Excavated levels (cm)	Proximal and complete flakes	Flake fragments	Chunks and nodules	Retouched artifacts*	Total	Mean weight per specimen (gr)	Summed weight (gr)
**02–12**	32	99	0	0	131	0.95 ±2.79	124.82
**12–22**	85	363	9	3	460	1.23 ±4.17	565.38
**22–32**	231	945	8	9	1,193	1.16 ±3.66	1,387.5
**32–42**	472	1,994	13	10	2,489	1.11 ±4.17	2,755.31
**42–52**	744	2,522	9	12	3,287	1.06 ±6.08	3,490.76
**52–62**	672	2,428	13	15	3,128	1.14 ±6.82	3,557.44
**62–72**	400	1,454	8	1	1,863	0.61 ±3.76	1,131.85
**72–82**	106	499	2	2	609	0.51 ±2.01	309.18
**82–92**	26	138	0	0	164	0.3 ±0.97	48.55
**92–102**	3	12	0	0	15	1.31 ±3.44	19.62
**102–110**	5	33	0	0	38	2.13 ±3.97	80.78
**Total**	2,776	10,487	62	52	13,377	1.01 ±5.23	13,471.19

Considers the whole assemblage.

*Includes tool classes and specimens with occasional removals.

**Fig 7:**
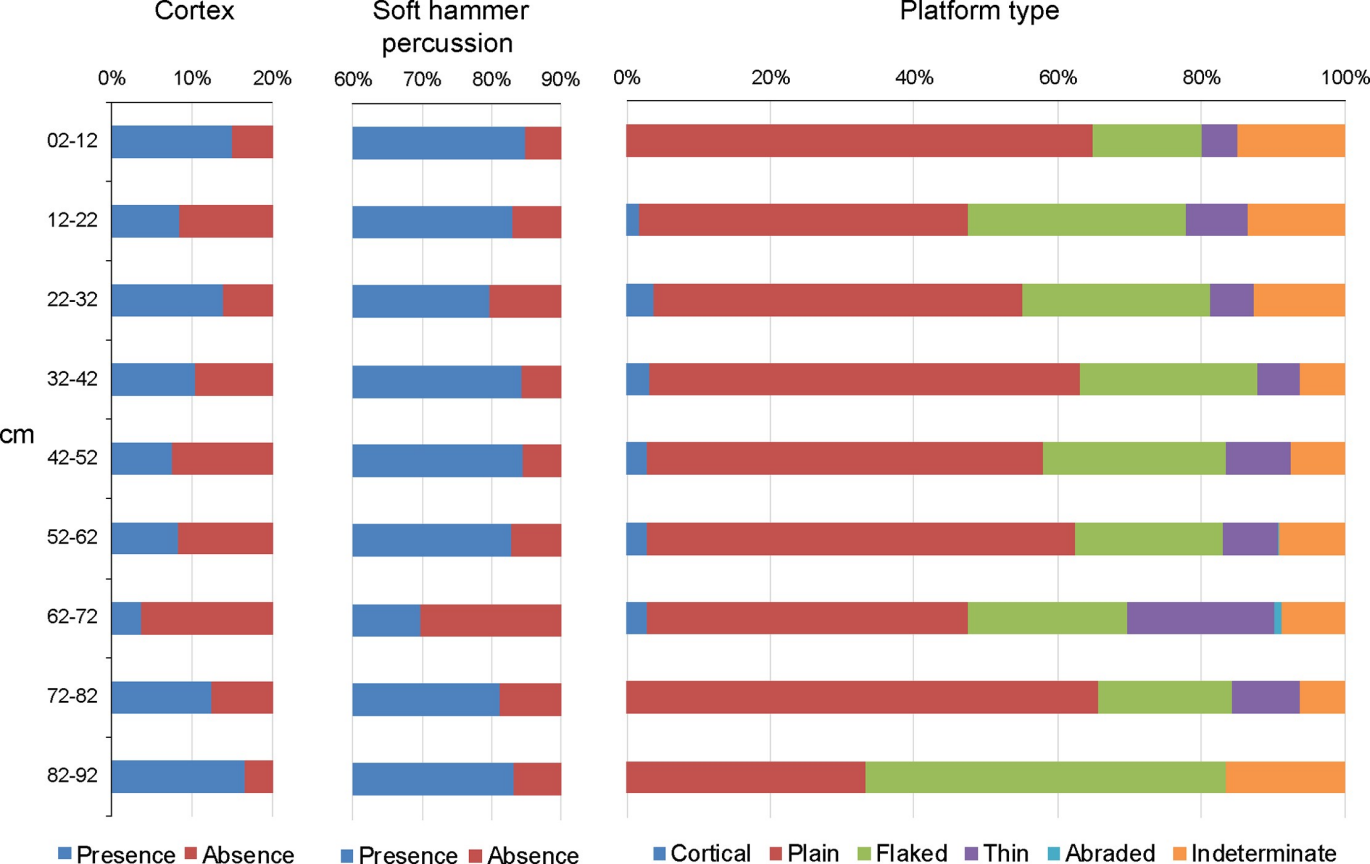
Main technological attributes of the lithic assemblage. Considers only complete and proximal fragments of piece plotted specimens.

A subsample from all the piece-plotted lithics (N = 3,938) was used for addressing the representation of attributes such as overall knapping quality, cortex presence, platform type, knapping technique, size distribution, and a tool/debitage classes. This subsample includes 1,701 complete and proximal sections of flakes which should be considered as the minimum number of lithic elements and the basis for all counts in other to avoid overrepresentation (Fig 7). Of all quartz specimens, 98.16% are crystalline. However hard, the degree of translucency is an indication of knapping quality for this toolstone [75]. Hence, we consider this assemblage to show a high degree of selectivity all throughout the excavated section. Cortex proportion varied between 3.72% and 16.67%. Soft hammer percussion, based on the absence of marked percussion bulb and of point of impact and on the overall flake morphology, dominated at each excavated level. Striking platforms include mainly flat and flaked types, while cortical and abraded platform types remained remarkably low. High fragmentation precluded from obtaining credible inferences regarding variability in debitage sizes. When considering all pieces bearing striking platform, size sorting indicated that 81.95% of flakes are <3cm in diameter, and that larger sizes (>5 cm in diameter) are not only infrequent, but also not represented in all excavated levels (S5 Fig). The conjunction of proportional values for these attributes suggests that knapping at the site was dominated by steps of formalization rather than the earliest stages of reduction. This is consistent with the very low frequency of core flakes and the absolute absence of cores (Table 3). In effect, the sample is comprised mainly of bifacial thinning flakes and smaller debitage classes (including edge trimming and pressure flakes), altogether 94.65%.

**Table 3:** Flake classes represented in the lithic assemblage.

Excavated levels (cm)	Debitage	Bifacial thinning flake	Core flake	Retouched flake	Undetermined pieces	Total
**02–12**	5 (25%)	13 (65%)	2 (10%)	0 (0%)	0 (0%)	20 (100%)
**12–22**	10 (16.95%)	45 (76.27%)	0 (0%)	2 (3.39%)	2 (3.39%)	59 (100%)
**22–32**	35 (21.21%)	118 (71.52%)	7 (4.24%)	2 (1.21%)	3 (1.82%)	165 (100%)
**32–42**	71 (20.11%)	261 (73.94%)	19 (5.38%)	0 (0%)	2 (0.57%)	353 (100%)
**42–52**	102 (21.94%)	343 (73.77%)	11 (2.37%)	0 (0%)	9 (1.94%)	465 (100%)
**52–62**	85 (22.08%)	280 (72.73%)	9 (2.34%)	2 (0.52%)	9 (2.34%)	385 (100%)
**62–72**	68 (31.63%)	137 (63.72%)	1 (0.47%)	1 (0.47%)	8 (3.73%)	215 (100%)
**72–82**	6 (18.75%)	25 (78.13%)	1 (3.13%)	0 (0%)	0 (0%)	32 (100%)
**82–92**	0 (0%)	5 (83.33%)	1 (16.67%)	0 (0%)	0 (0%)	6 (100%)
**92–102**	1 (100%)	0 (0%)	0 (0%)	0 (0%)	0 (0%)	1 (100%)
**Total**	383 (22.52%)	1227 (72.13%)	51 (2%)	7 (0.42%)	33 (1.95%)	1,701 (100%)

Considers only complete and proximal fragments of piece plotted specimens.

**Fig 8:**
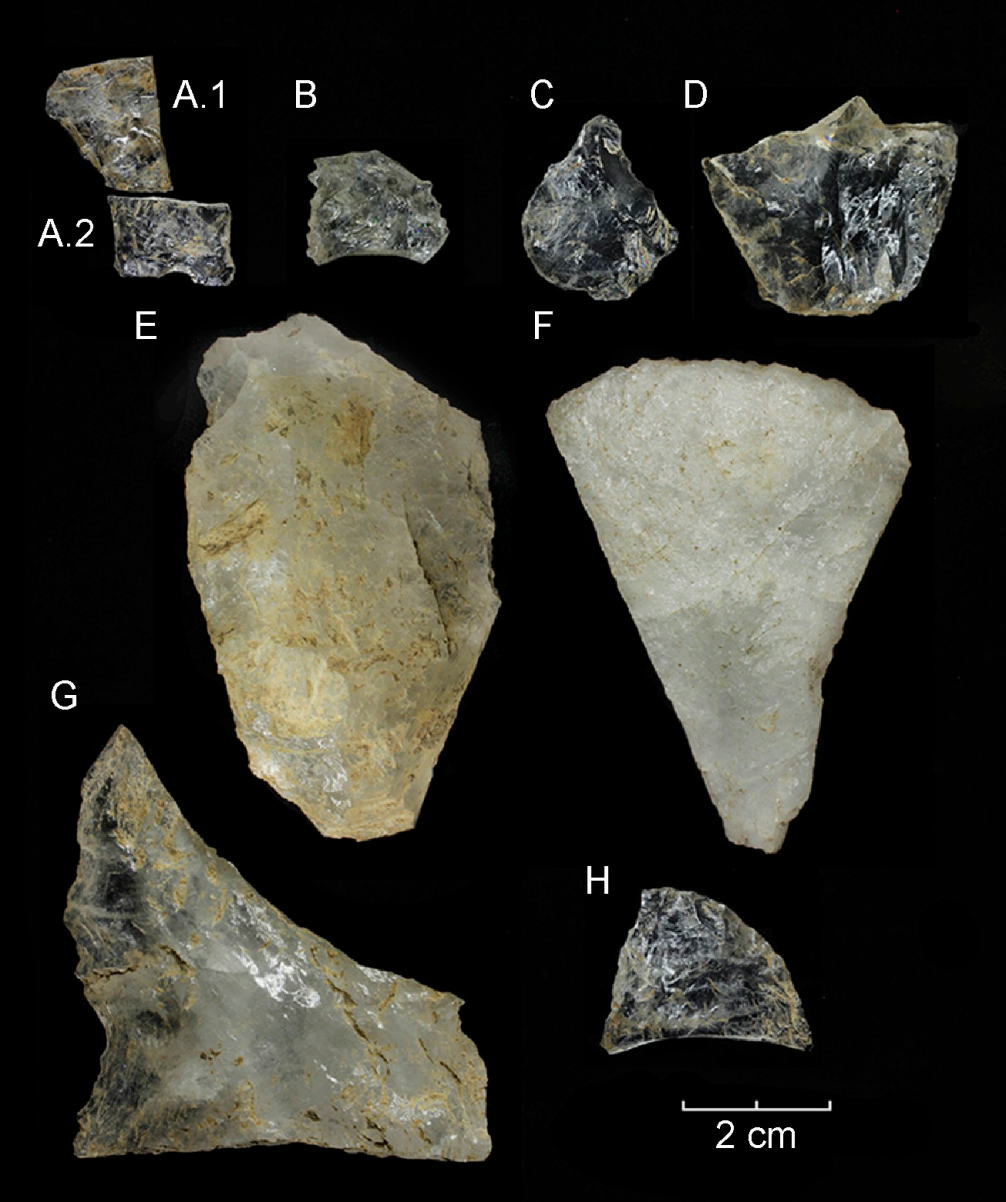
Selected quartz artifacts from Valiente. A.1. fishtail -type point preform mid-section (#304, unit A2, 52–62 cm), A.2. fishtail-type point preform stem (#305, unit A1, 52–62 cm), B. point fragment, mid-section (#RPA, exposed profile), C. lithic awl (#931, unit E2, 22–32 cm), D. bifacial blank (#184, unit A2, 42–52 cm), E. bifacial blank (#262, unit A2, 52–62 cm), F. scraper (#1080, unit E2, 22–32 cm), G. notched flake (#2528, unit B3, 42–52 cm), H. bimarginal cutting tool (#1775, unit C2, 42–52 cm).

As expected in the context of a quarry, retouched pieces, either bifacial or marginal, are only represented in very low frequency (Table 4). Fractured bifaces and bifacial preforms were recorded at almost all excavated levels. However, no final products have been recorded at this site. Successfully manufactured tools, including late stage bifaces and points, must have been taken away from the site. The only formal diagnostic pieces are the two conjoined fragments of a fishtail-type point preform (# 304 and 305) in an advanced stage of manufacture (Fig 8). This specimen indicates that, at least in some occupational events, knappers attempted the finish tools at the site. Other artifacts are minorly represented and include marginally retouched informal tools such as scrapers, knifes, and utilized flakes among others. These attest for other complementary activities carried out at the locale.

**Table 4:** Flake classes represented in the lithic assemblage.

Excavated levels (cm)	Biface and bifacial preform	Scraper	Sidescraper	Knife	Utilized flake	Awl	Total
**12–22**	0	1	1	0	0	0	2
**22–32**	2	2	0	1	0	1	6
**32–42**	5	0	0	1	2	0	8
**42–52**	5	2	0	3	2	0	12
**52–62**	6	0	0	0	4	0	10
**62–72**	1	0	0	0	0	0	1
**72–82**	1	0	1	0	0	0	2
**82–92**	0	0	0	0	0	0	0
**92–102**	0	0	0	0	0	0	0
**Test pit**	1	0	0	0	0	0	1
**Total**	16	5	2	5	8	1	42

Considers only piece plotted specimens.

#### Lithic taphonomy

A taphonomic analysis was implemented in a subsample of quartz lithics from excavation unit B2 to assess the magnitude of alteration traces across excavated levels [76]. Their presence, location and coverage were recorded for each of the specimens analyzed. Abrasion is the main recorded agent since it affects 99.18% of the cases. The lithic artifacts show various degrees of rounding, though medium grade abrasion on both sides occurs in 54.51%. These data are consistent with what is expected for dragging and surface exposure of the pieces. Body fractures occurred in 44.63% of the subsample (diagonal: 9.77%, longitudinal: 13.68% and transversal: 14.33%). Edge fractures are even more widespread (85.05%). Both patterns can be attributed to dragging and trampling, although it is not possible to rule out other potential alteration agents. Microchipping of the edges was observed in 100% of the pieces occurring on both sides. The combination of short, long and half-moon types was observed in 89.75%; mostly occurring isolated, but frequently superimposed. The microchipping of the flake scar ridges was recorded in 49.2% of the sample, however, its presence exceeds 80% in the dorsal face of specimens from the deepest excavated levels. Microchipping of edges and flake scar ridges are consistent with what is expected by dragging and trampling. A 32.38% of the analyzed pieces showed polishing similar to that observed in the pieces recorded along the slope. Other less represented traces are stretch marks (11.5%) and edge trituration (9.6%).

In summary, all specimens showed some degree of physical weathering, which is dominantly expressed on both sides of the pieces (96.45%). This suggests that lithics of all excavated levels inverted their position at least twice over time, which should be regarded as a measure of low stability of the artifacts. The taphonomic analysis of lithic material suggests that the main process of alteration was the dragging of flakes across the exposed surface of the slope, while trampling and other agents seem to have had less and only specific influence.

#### Faunal remains

A preliminary analysis indicated that the bone assemblage was mainly composed by small and heavily-burnt bone fragments, largely characterized as bone splinters [45]. The total studied assemblage is comprised by 536 bone specimens. Indeterminate bone splinters (87.31%) largely dominate this assemblage, followed by fragments of long bones (5.6%) and fragments of vertebrae (2.43%). Fragmentation is a major feature which limited diagnostic information. Bone pieces have a mean of 17.53 mm (sd: 6.84) value which remained similar across the excavated levels. Only a 16.23% of specimens are taxonomically identifiable beyond class level and only four were assigned to a species level (Table 5).

**Table 5:** Taxonomical identification of bone material across the excavated levels.

Excavated levels (cm)	12–22	22–32	32–42	42–52	52–62	62–72	72–82	82–92	92–102	Total	
Taxa	NISP	NISP	NISP	NISP	NISP	NISP	NISP	NISP	NISP	NISP	%NISP
**Bird**	1	2	1	1	1	0	0	0	0	6	1.12
**Mammalia**	4	7	34	98	168	91	34	6	1	443	82.65
**Mylodontidae**	0	0	0	1	0	0	0	0	0	1	0.19
**Arctiodactyla**	3	3	8	23	20	8	2	1	0	68	12.69
***Lama* sp.**	0	0	0	0	1	0	0	0	0	1	0.19
**Canidae**	1	0	0	2	0	1	0	0	0	4	0.75
***Lycalopex griseus***	0	0	0	2	2	0	0	0	0	4	0.75
**Rodentia**	0	2	0	1	1	4	0	1	0	9	1.68
**Total**	9	14	43	127	193	104	36	8	1	535	100

The assemblage presents evidence attributed to burning in 63.25% of the specimens, of which 3.54% are visibly scorched. The thermal alterations produced by burning are mostly black, which suggest a short time of fire exposure [77]. Probably, the high proportion of burnt bones is due precisely to the fact that non-combusted remains were less likely preserved. Though no hearths were recorded within the excavation, dispersed charcoal speckles suggest the occurrence of such structures in the proximity. Thus, we cannot rule out that the high frequency of burned material could be partly due to the intentional discard of bones into combustion structures or even their use as fuel [78]. However, SEM-EDS analyses on six samples showed traces of manganese and iron oxides which may have contributed to enhancing the black color and its extension (S3 Appendix). This further suggest that bones may have been temporarily in a water-saturated environment where the manganese oxide was likely to precipitate, although this may have occurred over a short period [65, 79].

The best represented taxonomic group is that of the order Artiodactyla. Skull fragments, vertebrae, long bones, phalanxes and astragalus are observed throughout the excavated levels, specially between 42 and 62 cm. Only one phalanx was attributed to *Lama* sp. Given that the only extant species of artiodactyl in the region is guanaco (*Lama guanicoe*), this is the most likely candidate to be represented at Valiente. Despite the existence of contemporaneous dates on bone material of *Equus* (*Amerhippus*) sp. and cf. *Palaeolama* sp. on the coast [80], bones of extinct Pleistocene mammals are robust, thicker and heavier, and visibly different to those fragments attributed to Mammalia at Valiente. Only one anomalously recent burnt dermal bone of Mylodontidae was recorded. Besides being suspected of vertical displacement in this particular case, dermal bones have been considered prone to migration within the deposits in which they occur [81]. Hence, there is no indication of intentional human involvement in its deposition. Also, rodent bones are among the unburnt material and consequently assumed to be incorporated through natural processes. The only taxa assigned to species level was the common fox (*Lycalopex griseus*) (S6 Fig). Fragments of long bones assigned to Canidae may also belong to this species. Anthropogenic marks are significantly infrequent in this assemblage, mainly because of high fragmentation, surface deterioration and burning. However, we were able to record sharp cutting marks on a burnt femur of *Lycalopex griseus* and one artiodactyl bone with international fragmentation and two cutmarks.

### Site context and human occupations of the Pleistocene-Holocene transition

Site formation processes and taphonomic observations on lithic, bone and charcoal material suugest that sedimentary particles, including archaeological material, moved downwards along the slope before their final burial. Downslope movement of materials has been acknowledged as a major post depositional mechanism in arid landscapes [82]. However, materials tend to accumulate in the base of the slopes [83]. As such, the Valiente site shows that despite such processes occurred, ages are in stratigraphic coherence and that the occurrence of even small and light particles in the excavated area indicate that relocation must have been spatially minor.

The time span of occupation of Valiente during the Pleistocene-Holocene transition is dated roughly between 12,630 and 11,320 cal BP (medians of earliest/latest ages). Based on radiocarbon dates form areas X and T we calculated a minimum of four occupational events in the site. These events have stratigraphic and depositional coherence. The initial occupation of the site occurred at 12,690–12,550 cal BP (see above), which is associated with the most significant amount of anthropogenic deposition of lithics, bone and charcoal. Another occupational event can be defined by averaging two dates (UGAMS 10293, BETA 279038) between 12,040–11,770 cal BP (10,686 ±19 BP), a third by combining two dates (UGAMS 10294, UGAMS 5886) between 11,760–11,400 cal BP (10,261 ±26 BP), and a fourth represented by a single date (UGAMS 7820) at 11,600–11,230 cal BP.

Activities conducted at the site inform of formalization stages in bifacial production which remained similar across the period represented. While the selection of nodules and early stages in blank procurement are completely absent in the excavated area, these must have occurred elsewhere within the quartz outcrop bounds. The presence of debris from thinning phases, edge trimming and retouch, altogether with bifacial fragments, including a fractured point preform, indicate that the whole operational sequence was carried out in the site at specific points in time. Primary selection of high-quality translucent quartz fragments indicates no noteworthy change in selectiveness with respect to quality throughout the occupations.

Few discarded informal edge tools and infrequent bone remains suggest other activities were carried out alongside quartz knapping. This is consistent with the expected high residential mobility for the earliest settlers of the region. Though the poor preservation of bone material precludes from conclusive inferences, it is interesting that this record suggests the consumption of extant taxa during the Pleistocene-Holocene transition.

## Discussion

The Pleistocene-Holocene transition is coincidental with the phase in which natural landscapes became the “taskscapes” for the earliest hunter-gatherers in the region [84], thereby encompassing cultural changes related to the organization of land use. Key places with desirable resources must have been redundantly visited after initial recognition and thereon provided the locales for social encounters. In such scenario, colonizing human groups should be expected to maintain bonds for a fluent exchange of information, thereby enhancing a low degree of territoriality [85]. Key to this organization was the way how groups planned their technology in attention to the regional raw material availability. Rather than a traditional territorial organization focused on localities, as expected for groups with durable ties to a given area, early settlers would be expected to organize landscape in association to key high-quality material resources, crucial for maintaining communication and exchange [86, 87].

At a local scale, the basal date of Valiente does not statistically overlap the age of Quebrada Santa Julia; yet they are within the confident range attributable to penecontemporaneous occupations [33]. Despite the differences between these sites, Quebrada Santa Julia being a brief stay campsite where crystal quartz was an exotic toolstone and Valiente a quarry/workshop where the initial stages of procurement and manufacture were carried out, some observations on technological behaviors suggest they should be regarded as integrated. However true that the lithic frequency at Valiente (N = 1,701) is twelve times the one in Santa Julia (N = 138), we attribute this difference to the fact that Valiente represents successive superimposed occupations at the raw material source. The thickness of the deposit and the higher depositional rate should not be regarded as indicative of higher intensity per se, but rather the averaged accumulation of visits of knappers to a fixed point where high-quality toolstone was available. Conversely, Quebrada Santa Julia did not only yielded evidence for quartz knapping, but also of tool manufacture and use of other toolstones of closer provenance [30].

Quartz crystal in Quebrada Santa Julia shows that core flakes, larger than any excavated at Valiente, and early stage bifaces were transported from the source location, since manufacture of edge and bifacial tools was performed at the site [32]. Incomplete operational sequences in the source, as shown by the Valiente assemblage, confirm the differential organization of activities across space. However, the presence of advanced stage preform rejects, both at the quarry/workshop and at the campsite, indicate that multiple procurement strategies may have coexisted. Despite the absence of change in quality selectivity along the sequence at Valiente, early Holocene occurrence of quartz in sites within >40 km from the source shows inferior qualities [35] (Table 6; Fig 1). Rarely, quartz in these sites shows the degree of translucency characteristic of Valiente and Quebrada Santa Julia. Lithic points are therefore thicker, coarser and with less clear edges.

**Table 6:** Quartz occurrence and frequencies in local late Pleistocene to early Holocene sites.

Site	Age/range (2σ cal BP)	Surface quartz occurrence	Stratigraphic quartz frequency	Distance to source (km)
**Quebrada Santa Julia**	12,990–12,730	None	35.2%	37
**Punta Ñagué**	12,430–11,250 to 10,380–10,200	Debitage	12.99%	40
**Punta Penitente**	10,190–9390	Point	0%	38
**Quereo Norte**	Undated	Point	3.88%	35
**Quereo Perfil**	9,400–9,030	None	Isolated flake	35
**Punta Purgatorio**	12,080–11,050 to 11,650–11,280	Debitage	0%	32
**Palo Colorado**	Undated	Point	ND	34
**Pichidangui**	9,000–8,240 to 8,590–8,440	Debitage	0.19%	34

Undated sites are presumed early Holocene in age based on regional typological attributes. ND: no data.

At a regional scale, the Pleistocene-Holocene transition experienced great environmental change with increasing dryness, diminishment in the proportion of tree taxa, lake basin encroaching, and megafauna disappearance across the landscape [49, 88–91]. The only site in central Chile sharing similar tool types in quartz crystal is Taguatagua 2 [92]. A confident occupational range between 11,720–11,210 cal BP indicates it is contemporaneous with the latest chronological range of Valiente [33]. However, Taguatagua 2 record is remarkably different, given that is characterized by a series of ordered piles of bones of the incomplete remains of at least nine butchered gomphotheres along with few finished tools and with no indication of local lithic production [33, 45, 92]. Yet, in the case of Taguatagua 2, crystal quartz fishtail points were recorded either broken (N = 1) or in their finished state (N = 2). Though we have not been able to establish if they belong to the variety of quartz, nonetheless, as in the case of Valiente and Quebrada Santa Julia, the evidence points out towards a high-quality toolstone selectivity [32].

Quartz quarries have been reported for the earliest inhabitants of Central America [93]. In the context of South America, quartz crystal has been proven to be used in the Pleistocene-Holocene transition [94] (Fig 1). Besides showing diverse degrees of knapping easiness, its translucency has been highlighted as a potential selective attribute [95]. At Quebrada Santa María (PV23-130) in the Chicama valley (La Libertad, Perú) quartz crystal comprises almost 70% of the lithic assemblage, including fishtail points (N = 8), bifacial fragments and marginally retouched tools associated with the remains of white deer, fish and reptiles [96, 97]. Similar assemblages have been reported for the neighbor sites at Quebrada Batán, featuring one age of 13,073–12,860 cal BP in association with crystal quartz [98]. Several quartz workshops, attributed to the Pleistocene Holocene transition based on typological attributes, have been recorded in the same region, though no direct radiocarbon dates have been obtained from them [99]. Quartz fishtail points have also been recorded at Amigo Oeste (N = 5; 3.52%) in Meseta del Somuncurá (Río Negro, Argentina) and at several sites in Uruguay [95, 100]. However, formal tools on translucent quartz confidently constrained within the time ranges of the Pleistocene-Holocene transition are few. Quartz crystal utilization has also been described for butchering sites such as La Moderna (Pampas, Argentina) where remains of glyptodont (*Doedicurus clavicaudatus*) were excavated in association with tools (N = 16) and debitage (>2000 pieces) procured from a local source (~1 km) [101]. Besides the sites discussed here, fishtail artifacts manufactured in quartz were recorded at a similar latitude in the Atlantic rim at the Tigre site (Uruguay) with ages between 12,640 and 12,320 cal BP [102, 103]. All these evidences lie within the expected spatial and chronological range distributions of fishtail points [29, 104, 105]. However, no workshop evidence has been described so far within well constrained chronological ranges.

The archaeology of early workshops is not common across America. Despite their recognized pivotal role in the technological organization of mobile hunter-gatherers, detailed analyses and comprehensive radiocarbon chronologies in such sites are largely missing. The Gault site is one workshop in Texas whose deposits may represent a handful of knapping episodes over a relatively short time span as suggested by refitting and intrasite spatial analysis [12]. The absence of exotic raw materials makes it singular in terms of contemporary occupations in North America. As in the case with the Gault site, Valiente does not occur along a major watercourse, thus suggesting its occupants were familiar to the outcrop when visiting it, rather than exploring along least costly paths such as the coast or the main east-west rivers and creeks. Valiente site redundancy does not support its consideration as an occasional stop for replenishing depleted toolkits, but rather as a well-known location accessed by hunter-gatherers through planned visits.

The dominance in the use of local raw materials has been indicated as an argument supporting a detailed knowledge of the landscapes among early occupants of northern South America [106]. Several terminal Pleistocene sites in South America show distant linkages such as the one recorded for Santa Julia and Valiente or even in greater distances. For instance, Quebrada Jaguay 280 and Cuncaicha are coastal and highland locations in Arequipa (Perú), respectively, which have been acknowledged to have been articulated as the end-members of a settlement system as indicated by overlapping ages and the presence of obsidian from the same highland source [107, 108]. In this regard, patterned lithic procurement behavior should not be regarded as a process occurring later in time but already taking place in some areas by the Pleistocene-Holocene transition. Landscape learning regarding the distribution of key lithic resources may have occurred fast and possible enabled by the visibility of obtrusive high-quality toolstones, such as obsidian or quartz crystal.

## Conclusions

The Valiente quarry/workshop has yielded conclusive evidence for the procurement of high-quality quartz crystal since the terminal Pleistocene and extending during 1,300 years of repeated short-term visits. Despite the singularities of a slope deposit in an arid environment prone to erosion and surface dragging, local topographic features made continuous sedimentation possible, thus producing a stratigraphically ordered deposit. Organic materials (bone and charcoal) were recorded in direct association with lithics attesting early stage production of bifacial tools and hence they account for a previously understudied type of site in the archaeology of early South Americans. Quarrying behavior observed at this site was most likely associated to penecontemporaneous campsites such as Quebrada Santa Julia and Taguatagua 2 which have produced artifacts in quartz crystal [30, 92]. Other sites in the region, as early as the evidence presented here (e.g. Taguatagua 1) have no quartz evidence, yet they provide a broader picture of differential activities across an evolving landscape [32, 33, 109]. In the broader South America, sites with fishtail-type points suggest that quartz crystal, along with other attractive raw materials, was selected based on attributes beyond knapping easiness or mechanical fracture properties, and that translucency and color may have played a key role in selection [24, 95, 110, 111].

## Supporting information

S1 Fig Topographic map of the Valiente site. (PDF)

S2 Fig Frequency of piece-plotted lithic specimens by depth from artificial datum. (PDF)

S3 Fig Lithic distributions per excavated unit of area X partitioned by level. (PDF)

S4 Fig 3D reconstruction of piece-plotted specimens from area X. Vertical (depth) axis is exaggerated.
(PDF)

S5 Fig Flake size diameter intervals per excavated level. Intervals in X axis represent 1 cm increase.
(PDF)

S6 Fig Selected burnt bone remains from the Valiente site. A. left astragalus, *Lycalopex griseus*; B. radius, *Lycalopex griseus*; C. distal end of femur, *Lycalopex griseus*; D. vertebra fragment, Artiodactyla; E. indeterminate long bone fragment, Mammalia.
(PDF)

S1 Appendix Detailed stratigraphy of Area X at the Valiente site. (PDF)

S2 Appendix The bioarchaeological context of Area U at the Valiente site. (PDF)

S3 Appendix Scanning Electron Microscopy of faunal material of Area X at the Valiente site. (PDF)

## References

[1] Dillehay TD. The Settlement of the Americas: a New Prehistory New York: Basic Books; 2002.

[2] Odell GH. A North American perspective on recent archaeological stone tool research. Palimpsesto Revista de Arqueología. 1993;3:109–22.

[3] Bamforth DB. The Windy Ridge quartzite quarry: Hunter-gatherer mining and hunter-gatherer land use on the North American Continental Divide. World Archaeology. 2006;38(1):511–27.

[4] Bamforth DB. Quarries in context: a regional perspective on lithic procurement In: Arnold J, editor. Stone Tool Procurement, Production, and Distribution in California Prehistory. Los Angeles: Institute of Archaeology, University of California; 1992 p. 131–50.

[5] Beck C, Taylor AK, Jones GT, Fadem CM, Cook CR, Millward SA. Rocks are heavy: transport costs and Paleoarchaic quarry behavior in the Great Basin. Journal of Anthropological Archaeology. 2002;21(4):481–507.

[6] Binford LR. The archaeology of place. Journal of Anthropological Archaeology. 1982;1(1):5–31. 10.1016/0278-4165(82)90006-X.

[7] Núñez L. Los cazadores tempranos de los Andes meridionales: Evaluación cronólogica de las industrias líticas del norte de Chile. Boletín de Antropología Americana. 1980;2:87–120.

[8] Mayer-Oakes WJ. El Inga: A Paleo-Indian site in the sierra of northern Ecuador. Transactions of the American Philosophical Society. 1986;76(4):i–235. 10.2307/1006466

[9] Barkai R, Gopher A. Changing the face of the earth: Human behavior at Sede Ilan, an extensive Lower–Middle Paleolithic quarry site in Israel In: Adams B, Blades B, editors. Lithic Materials and Paleolithic Societies. Oxford: Blackwell Publishers; 2009 p. 174–85.

[10] Wragg Sykes RM, Delvigne V, Fernandes P, Piboule M, Lafarge A, Defive E, et al “Undatable, unattractive, redundant”? The Rapavi silcrete source, Saint-Pierre-Eynac (Haute-Loire, France): Challenges studying a prehistoric quarry-workshop in the Massif Central mountains. Journal of Archaeological Science: Reports. 2017;15:587–610. 10.1016/j.jasrep.2017.07.022.

[11] Ericson J. Toward the analysis of lithic production systems In: Ericson J, Purdy B, editors. Prehistoric Quarries and Lithic Production. New York: Cambridge University Press; 1984 p. 1–9.

[12] Waters MR, Pevny CD, Carlson DL, Dickens WA, Minchak SA, Smallwood AM, et al Clovis Lithic Technology: Investigation of a Stratified Workshop at the Gault Site, Texas: Texas A&M University Press; 2011.

[13] Waters MR, Forman SL, Jennings TA, Nordt LC, Driese SG, Feinberg JM, et al The Buttermilk Creek Complex and the Origins of Clovis at the Debra L. Friedkin Site, Texas. Science. 2011;331(6024):1599 10.1126/science.1201855 21436451 10.1126/science.1201855

[14] Smallwood AM. Context and spatial organization of the Clovis assemblage from the Topper site, South Carolina. Journal of Field Archaeology. 2015;40(1):69–88. 10.1179/0093469014Z.000000000106

[15] Loyola R, Cartajena I, Núñez L, López P. Moving into an arid landscape: Lithic technologies of the Pleistocene–Holocene transition in the high-altitude basins of Imilac and Punta Negra, Atacama Desert. Quaternary International. 2018;473:206–24. 10.1016/j.quaint.2017.10.010.

[16] Herrera KA. La industria lítica bifacial del sitio en cantera Chipana-1 Conocimiento y técnica de los grupos humanos del Desierto de Atacama, norte de Chile al final del Pleistoceno. Taladoire E, editor. Oxford: Archaeopress; 2018. 117 p.

[17] Pelegrin J, Chauchat C. Tecnologia y función de las puntas de Paiján: El aporte de la experimentación. Latin American Antiquity. 1993;4(4):367–82. Epub 2017/01/20. 10.2307/972073

[18] Stern CR. Obsidian sources and distribution in Patagonia, southernmost South America. Quaternary International. 2018;468:190–205. 10.1016/j.quaint.2017.07.030.

[19] Nami HG, Stanford DJ. Dating the peopling of northwestern South America: An AMS date from El Inga site, highland Ecuador. PaleoAmerica. 2016;2(1):60–3. 10.1080/20555563.2016.1139793

[20] Flegenheimer N, Mazzia N, Weitzel C. Landscape and rocks in the east-central portion of the Tandilia range (Buenos Aires Province, Argentina). PaleoAmerica. 2015;1(2):163–80. 10.1179/2055556315Z.00000000017

[21] Sandweiss DH, Rademaker KM. El poblamiento del sur peruano: costa y sierra. Boletín de Arqueología PUCP. 2011;15:275–93.

[22] Nami HG. Consideraciones tecnológicas preliminares sobre los artefactos líticos de Cerro de los Burros (Maldonado, Uruguay). Comunicaciones Antropológicas de los Museos Nacionales de Historia Natural y Antropología. 2001;3(21):1–24.

[23] Méndez C, Stern CR, Nuevo Delaunay A, Reyes O, Gutiérrez F, Mena F. Spatial and temporal distributions of exotic and local obsidians in Central Western Patagonia, southernmost South America. Quaternary International. 2018;468:155–68. 10.1016/j.quaint.2017.08.062

[24] Flegenheimer N, Bayón C, Valente M, Baeza J, Femenías J. Long distance tool stone transport in the Argentine Pampas. Quaternary International. 2003;109–110:49–64. 10.1016/S1040-6182(02)00202-1.

[25] Rademaker K, Reid DA, Bromley GRM. Connecting the dots: least-cost analysis, paleogeography, and the search for Paleoindian sites in southern highland Peru In: White DA, Surface-Evans S, editors. Least Cost Analysis of Social Landscapes: Archaeological Case Studies. Salt Lake City: University of Utah Press; 2012 p. 32–45.

[26] Dillehay TD, Ocampo C, Saavedra J, Sawakuchi AO, Vega RM, Pino M, et al New Archaeological Evidence for an Early Human Presence at Monte Verde, Chile. PLOS ONE. 2015;10(11):e0141923 10.1371/journal.pone.0141923 26580202 10.1371/journal.pone.0141923 PMC4651426

[27] Politis GG, Gutiérrez MA, Rafuse DJ, Blasi A. The Arrival of Homo sapiens into the Southern Cone at 14,000 Years Ago. PLOS ONE. 2016;11(9):e0162870 10.1371/journal.pone.0162870 27683248 10.1371/journal.pone.0162870 PMC5040268

[28] Dillehay TD, Ramirez C, Pino M, Collins MB, Rossen J, Pino-Navarro JD. Monte Verde: seaweed, food, medicine, and the peopling of South America. Science. 2008;320(5877):784–6. Epub 2008/05/10. 10.1126/science.1156533 . 18467586 10.1126/science.1156533

[29] Méndez C. Terminal Pleistocene/early Holocene 14C dates form archaeological sites in Chile: Critical chronological issues for the initial peopling of the region. Quaternary International. 2013;301:60–73. 10.1016/j.quaint.2012.04.003

[30] Jackson D, Méndez C, Seguel R, Maldonado A, Vargas G. Initial occupation of the Pacific coast of Chile during late Pleistocene times. Current Anthropology. 2007;48(5):725–31. 10.1086/520965 WOS:000249505800007.

[31] Jackson D, Méndez C, Aspillaga E. Human remains directly dated to the Pleistocene-Holocene transition support a marine diet for early settlers of the Pacific coast of Chile. Journal of Island & Coastal Archaeology. 2012;7(3):363–77.

[32] Méndez C. Los Primeros Andinos Tecnología Lítica de los Habitantes del Centro de Chile Trece Mil Años Atrás. Lima: Fondo Editorial Pontificia Universidad Católica del Perú; 2015. 252 p.

[33] Méndez C, Jackson D. Terminal Pleistocene lithic technology and use of space in central Chile. Chungara, Revista de Antropología Chilena. 2015;47(1):53–65.

[34] Ortega C, Vargas G, Rutllant JA, Jackson D, Méndez C. Major hydrological regime change along the semiarid western coast of South America during the early Holocene. Quaternary Research. 2012;78(03):513–27. 10.1016/j.yqres.2012.08.002

[35] Méndez C, Jackson D. Procuring quartz crystal in latest-Pleistocene/early-Holocene sites in Northern Semiarid and Mediterranean-Central, Chile In: Miotti L, Salemme M, Flegenheimer N, Goebel T, editors. Southbound: Late Pleistocene Peopling of Latin America. College Station: Center for the Study of the First Americans; 2012 p. 79–82.

[36] Rivano S, Sepúlveda P. Hoja Illapel, Región Coquimbo Carta Geológica de Chile. Santiago: SERNAGEOMIN; 1991.

[37] Méndez C, Jackson D, Seguel R, Nuevo Delaunay A. Early high-quality lithic procurement in the semiarid north of Chile. Current Research in the Pleistocene. 2010;27:19–21.

[38] Garreaud RD, Vuille M, Compagnucci R, Marengo J. Present-day South American climate. Palaeogeography, Palaeoclimatology, Palaeoecology. 2009;281(3):180–95. 10.1016/j.palaeo.2007.10.032.

[39] Romero H. Geografía de los Climas Geografía de Chile. Santiago: Instituto Geográfico Militar; 1985.

[40] Luebert F, Pliscoff P. Sinopsis bioclimática y vegetacional de Chile Santiago: Editorial Universitaria; 2006.

[41] Montecinos A, Aceituno P. Seasonality of the ENSO-related rainfall variability in central Chile and associated circulation anomalies. Journal of Climate. 2003;16(2):281–96. 10.1175/1520-0442(2003)016<0281:SOTERR>2.0.CO;2

[42] Niemeyer H, Cereceda P. Hidrografía, Geografía de Chile Santiago: Instituto Geográfico Militar; 1984.

[43] Romero H. Geografía de los Climas. Geografía de Chile Santiago: Instituto Geográfico Militar; 1985.

[44] Quintanilla V. Biogeografía. Geografía de Chile. Santiago: Instituto Geográfico Militar; 1983.

[45] Jackson D, Méndez C, Núñez L, Jackson D. Procesamiento de fauna extinta durante la transición Pleistoceno-Holoceno en el centro-norte de Chile. Boletín de Arqueología PUCP. 2011;15:315–36.

[46] Núñez L, Varela J, Casamiquela R, Villagrán C. Reconstrucción multidisciplinaria de la ocupación prehistórica de Quereo, centro de Chile. Latin American Antiquity. 1994;5(2):99–118.

[47] López P. Tafonomía de los mamíferos extintos del Pleistoceno Tardío de la costa meridional del Semiárido de Chile (IV Región-32° Latitud S). Alcances culturales y paleoecológicos. Chungara, Revista de Antropología Chilena. 2007;39(1):69–86.

[48] Villagrán C, Varela J. Palynological evidence for increased aridity on the central Chilean coast during the Holocene. Quaternary Research. 1990;34(2):198–207. 10.1016/0033-5894(90)90031-F

[49] Maldonado A, De Porras ME, Zamora A, Rivadeneira M, Abarzúa AM. El escenario geográfico y paleoambiental de Chile In: Falabella F, Uribe M, Sanhueza L, Aldunate C, Hidalgo J, editors. Prehistoria en Chile Desde sus Primeros Habitantes hasta los Incas. Santiago: Editorial Universitara; 2016 p. 23–70.

[50] Maldonado A, Méndez C, Ugalde P, Jackson D, Seguel R, Latorre C. Early Holocene climate change and human occupation along the semiarid coast of north-central Chile. Journal of Quaternary Science. 2010;25(6):985–8. 10.1002/jqs.1385

[51] Kim J-H, Schneider RR, Hebbeln D, Müller PJ, Wefer G. Last deglacial sea-surface temperature evolution in the Southeast Pacific compared to climate changes on the South American continent. Quaternary Science Reviews. 2002;21(18):2085–97. 10.1016/S0277-3791(02)00012-4.

[52] Kaiser J, Schefuß E, Lamy F, Mohtadi M, Hebbeln D. Glacial to Holocene changes in sea surface temperature and coastal vegetation in north central Chile: high versus low latitude forcing. Quaternary Science Reviews. 2008;27(21):2064–75. 10.1016/j.quascirev.2008.08.025.

[53] Maldonado A, Villagrán C. Climate variability over the last 9900 cal yr BP from a swamp forest pollen record along the semiarid coast of Chile. Quaternary Research. 2006;66(2):246–58. Epub 2017/01/20. 10.1016/j.yqres.2006.04.003

[54] Bronk Ramsey C. Deposition models for chronological records. Quaternary Science Reviews. 2008;27(1):42–60. 10.1016/j.quascirev.2007.01.019.

[55] Bronk Ramsey C. Bayesian analysis of radiocarbon dates. Radiocarbon. 2009;51(1):337–60. Epub 2016/07/18. 10.1017/S0033822200033865

[56] Hogg AG, Hua Q, Blackwell PG, Niu M, Buck CE, Guilderson TP, et al SHCal13 Southern Hemisphere calibration, 0–50,000 years cal BP. Radiocarbon. 2013;55(4):1889–903.

[57] Ward GK, Wilson SR. Procedures for comparing and combining radiocarbon age determinations: a critique. Archaeometry. 1978;20(1):19–31. 10.1111/j.1475-4754.1978.tb00208.x

[58] Holdaway S, Stern N. A Record in Stone: The Study of Australia's Flaked Stone Artefacts Canberra: Aboriginal Studies Press; 2004. 376 p.

[59] Andrefsky W. Lithics: Macroscopic Approaches to Analysis Cambridge: Cambridge University Press; 1998. 301 p.

[60] Petraglia MD, Potts R. Water flow and the formation of Early Pleistocene artifact sites in Olduvai Gorge, Tanzania. Journal of Anthropological Archaeology. 13:228–54.

[61] Borrazzo K. Tafonomía lítica y pseudoartefactos: el caso de la península El Páramo (Tierra del Fuego, Argentina). Intersecciones en Antropología. 2011;12:261–73.

[62] Burroni D, Donahue RE, Pollard AM, Mussi M. The surface alteration features of flint artefacts as a record of environmental processes. Journal of Archaeological Science. 2002;29(11):1277–87.

[63] Binford LR. Bones: Ancient Men and Modern Myths New York: Academic Press; 1981. 322 p.

[64] Grayson DK. Quantitative Zooarchaeology: Topics in the Analysis of Archaeological Faunas Orlando: Academic Press; 1984. 202 p.

[65] Lyman L. Vertebrate Taphonomy Cambridge: Cambridge University Press; 1994. 524 p.

[66] Bosch MD, Nigst PR, Fladerer FA, Antl-Weiser W. Humans, bones and fire: Zooarchaeological, taphonomic, and spatial analyses of a Gravettian mammoth bone accumulation at Grub-Kranawetberg (Austria). Quaternary International. 2012;252:109–21. 10.1016/j.quaint.2011.08.019.

[67] Rivano S, Sepúlveda P, Herve M, Puig A. Geocronología K-Ar de las rocas intrusivas entre los 31°-32°S, latitud sur, Chile. Revista Geológica de Chile. 1985;24:27–32.

[68] Gajardo A, López MC. Yacimientos de rocas y minerales industriales de la IV región de Coquimbo, Escala 1:500.000 Santiago: SERNAGEOMIN; 2004. 16 p.

[69] Church T. Lithic Resource Studies: A Sourcebook for Archaeologists Tulsa: University of Tulsa; 1994.

[70] Driscoll K. Vein quartz in lithic traditions: an analysis based on experimental archaeology. Journal of Archaeological Science. 2011;38(3):734–45. 10.1016/j.jas.2010.10.027.

[71] Jackson D, Galarce P, Seguel R. Asentamiento del Complejo Huentelauquén en Caimanes: relaciones entre valles interiores y costa. Boletín de la Sociedad Chilena de Arqueología. 2014;43/44:23–4.

[72] Hérail G. Formación de los pedimentos: aspecto histórico de una investigación Congreso Geológico Chileno 14; La Serena: Colegio de Geólogos de Chile A.G.; 2015.

[73] Morrow T. Lithic refitting and archaeological site formation processes. A case study from the Twin Ditch site, Greene County, Illinois In: Odell G, editor. Stone Tools: Theoretical Insights into Human Prehistory. New York: Plenum Press; 1996 p. 345–73.

[74] Cahen D, Keeley L, Van Noten F. Stone tools, toolkits, and human behavior in prehistory. Current Anthropology. 1979;20(4):661–72.

[75] Galarce P. Cazadores-recolectores tempranos en la costa sur del semiárido: aprovisionamiento y procesamiento de recursos líticos Santiago: Universidad de Chile; 2004.

[76] Méndez V. Historias depositacionales de conjuntos líticos en la Transición Pleistoceno-Holoceno en el sitio Valiente, Provincia del Choapa Santiago: Universidad de Chile; 2015.

[77] Joly D, March R, Martínez G. Les os brûlés de Paso Otero 5: un témoignage possible de l’utilisation de l’os comme combustible par des chasseurs-cueilleurs de la fin du Pléistocène en Argentine. Archeosiences, Revue d’Archéométrie. 2005;29:83–93.

[78] Théry-Parisot I. Fuel management (bone and wood) during the Lower Aurignacian in the Pataud Rock Shelter (Lower Palaeolithic, Les Eyzies de Tayac, Dordogne, France). Contribution of Experimentation. Journal of Archaeological Science. 2002;29:1415–21. 10.1006/jasc.2001.0781

[79] Martin FM. Tafonomía de la Transición Pleistoceno-Holoceno en Fuego-Patagonia Interacción entre Humanos y Carnívoros y su Importancia como Agentes en la Formación del Registro Fósil. Punta Arenas: Universidad de Magallanes; 2013. 406 p.

[80] Méndez C, Jackson D, Seguel R. Equus and Palaeolama direct 14C ages at Las Monedas site, Semiarid North of Chile. Current Research in the Pleistocene. 2011;28:107–9.

[81] López-Mendoza P, Mena-Larraín F. Extinct ground sloth dermal bones and their role in the taphonomic research of caves: the case of Baño Nuevo-1 (Andean Central Patagonia, Chile). Revista Mexicana de Ciencias Geológicas. 2011;28(3):519–32.

[82] Rick JW. Downslope movement and archaeological intrasite spatial analysis. American Antiquity. 1976;41(2):133–44. 10.2307/279164

[83] Goldberg P, Macphail R. Practical and Theoretical Geoarchaeology Malden, MA; Oxford: Blackwell Publishing; 2006. 454 p.

[84] Gamble C. The Palaeolithic Societies of Europe Cambridge, U.K.; New York: Cambridge University Press; 1999. 505 p.

[85] Kelly RL. Colonization of new land by hunter-gatherers: expectations and implications based on ethnographic data In: Rockman M, Steele J, editors. Colonization of Unfamiliar Landscapes: the Archaeology of Adaptation. London: Routledge; 2003 p. 44–59.

[86] Kelly RL, Todd LC. Coming into the country: Early Paleoindian hunting and mobility. American Antiquity. 1988;53(2):231–44. 10.2307/281017

[87] Gillespie JD. Enculturing an unknown world: Caches and Clovis landscape ideology. Canadian Journal of Archaeology. 2007;31(2):171–89.

[88] Valero-Garcés BL, Jenny B, Rondanelli M, Delgado-Huertas A, Burns SJ, Veit H, et al Palaeohydrology of Laguna de Tagua Tagua (34° 30′ S) and moisture fluctuations in Central Chile for the last 46 000 yr. Journal of Quaternary Science. 2005;20(7‐8):625–41. 10.1002/jqs.988

[89] Heusser CJ. Ice age vegetation and climate of subtropical Chile. Palaeogeography, Palaeoclimatology, Palaeoecology. 1990;80(2):107–27. 10.1016/0031-0182(90)90124-P.

[90] Barnosky AD, Lindsey EL. Timing of Quaternary megafaunal extinction in South America in relation to human arrival and climate change. Quaternary International. 2010;217(1):10–29. 10.1016/j.quaint.2009.11.017.

[91] Borrero LA. The elusive evidence: The archeological record of the South American extinct megafauna In: Haynes G, editor. American Megafaunal Extinctions at the End of the Pleistocene. Dordrecht: Springer; 2009 p. 145–68.

[92] Núñez L, Varela J, Casamiquela R, Schiappacasse V, Niemeyer H, Villagrán C. Cuenca de Taguatagua en Chile: el ambiente del Pleistoceno superior y ocupaciones humanas. Revista Chilena de Historia Natural. 1994;67:503–19.

[93] Pearson GA. First report of a newly discovered Paleoindian quarry site on the isthmus of Panama. Latin American Antiquity. 2003;14(3):311–22. 10.2307/3557562

[94] Roosevelt AC, da Costa ML, Machado CL, Michab M, Mercier N, Valladas H, et al Paleoindian cave dwellers in the Amazon: the peopling of the Americas. Science. 1996;272(5260):373–84.

[95] Nami HG. Crystal quartz and fishtail projectile points: considerations and raw-material selection by Paleo South Americans. Current Research in the Pleistocene. 2009;26:9–12.

[96] Briceño J. Quebrada Santa María: las puntas en cola de pescado y la antigüedad del hombre en Sudamérica. Boletín de Arqueología PUCP. 1999;3:19–39.

[97] Chauchat C, Briceño J. Paiján and fishtail points from Quebrada Santa María, North Coast of Perú. Current Research in the Pleistocene. 1998;15:10–1.

[98] Maggard GJ. The El Palto phase of northern Perú: cultural diversity in the late Pleistocene-early Holocene. Chungara, Revista de Antropología Chilena. 2015;47(1):25–40.

[99] Becerra R. Circulación y transformación de materias primas: el caso del Paijanense en el valle de Chicama (11.000–7000 a.p.) Boletín de Arqueología PUCP 1999;3.

[100] Hermo D, Terranova E, Miotti L. Tecnología y uso de materias primas en puntas cola de pescado de la meseta de Somuncurá (Provincia de Río Negro, Argentina). Chungara, Revista de Antropología Chilena. 2015;47(1): 101–15.

[101] Messineo PG, Gutierrez MA, Politis GG. Las primeras poblaciones indígenas de la región. In: Endere ML, Prado JL, editors. Patrimonio, ciencia y comunidad Su abordaje en los partidos de Azul, Olavarría y Tandil Olavarría: Universidad Nacional del Centro de la Provincia de Buenos Aires; 2009 p. 143–64.

[102] Suárez R, Piñeiro G, Barceló F. Living on the river edge: The Tigre site (K-87) new data and implications for the initial colonization of the Uruguay River basin. Quaternary International. 2018;473:242–60. 10.1016/j.quaint.2017.08.024.

[103] Suárez R. The Paleoamerican occupation of the plains of Uruguay: Technology, adaptations, and mobility. PaleoAmerica. 2015;1(1):88–104. 10.1179/2055556314Z.00000000010

[104] Prates L, Politis G, Steele J. Radiocarbon chronology of the early human occupation of Argentina. Quaternary International. 2013;301:104–22. 10.1016/j.quaint.2013.03.011.

[105] Weitzel C, Mazzia N, Flegenheimer N. Assessing Fishtail points distribution in the southern Cone. Quaternary International. 2018;473:161–72. 10.1016/j.quaint.2018.01.005.

[106] Gnecco C, Aceituno J. Early humanized landscapes in northern South America In: Morrow J, Gnecco C, editors. Paleoindian Archaeology A Hemispheric Perspective. Gainesville: University Press of Florida; 2006 p. 86–104.

[107] Rademaker K, Hodgins G, Moore K, Zarrillo S, Miller C, Bromley GRM, et al Paleoindian settlement of the high-altitude Peruvian Andes. Science. 2014;346(6208):466 10.1126/science.1258260 25342802 10.1126/science.1258260

[108] Sandweiss DH, McInnis H, Burger RL, Cano A, Ojeda B, Paredes R, et al Quebrada Jaguay: Early South American Maritime Adaptations. Science. 1998;281(5384):1830 9743490 10.1126/science.281.5384.1830

[109] Montané J. Paleo-Indian Remains from Laguna de Tagua Tagua, Central Chile. Science. 1968;161(3846):1137 10.1126/science.161.3846.1137 17812286 10.1126/science.161.3846.1137

[110] Nami HG. Silcrete as a valuable resource for stone tool manufacture and its use by Paleo-American hunter–gatherers in southeastern South America. Journal of Archaeological Science: Reports. 2017;15:539–60. 10.1016/j.jasrep.2016.05.003.

[111] Súarez R. Movilidad, acceso y uso de ágata traslúcida por los cazadores-recolectores tempranos durante la transición Pleistoceno-Holoceno en el norte de Uruguay (ca. 11,000–8500 a.P.). Latin American Antiquity. 2011;22(3):359–83.

