# Supplementary figures and images for: Late Pleistocene to early Holocene high-quality quartz crystal procurement from the Valiente quarry workshop site (32°S, Chile, South America)
Source: PLoS One. 2018 Nov 29;13(11):e0208062. doi: 10.1371/journal.pone.0208062 (PMC6264839; doi:10.1371/journal.pone.0208062)

# TOPOGRAPHY OF THE VALIENTE SITE

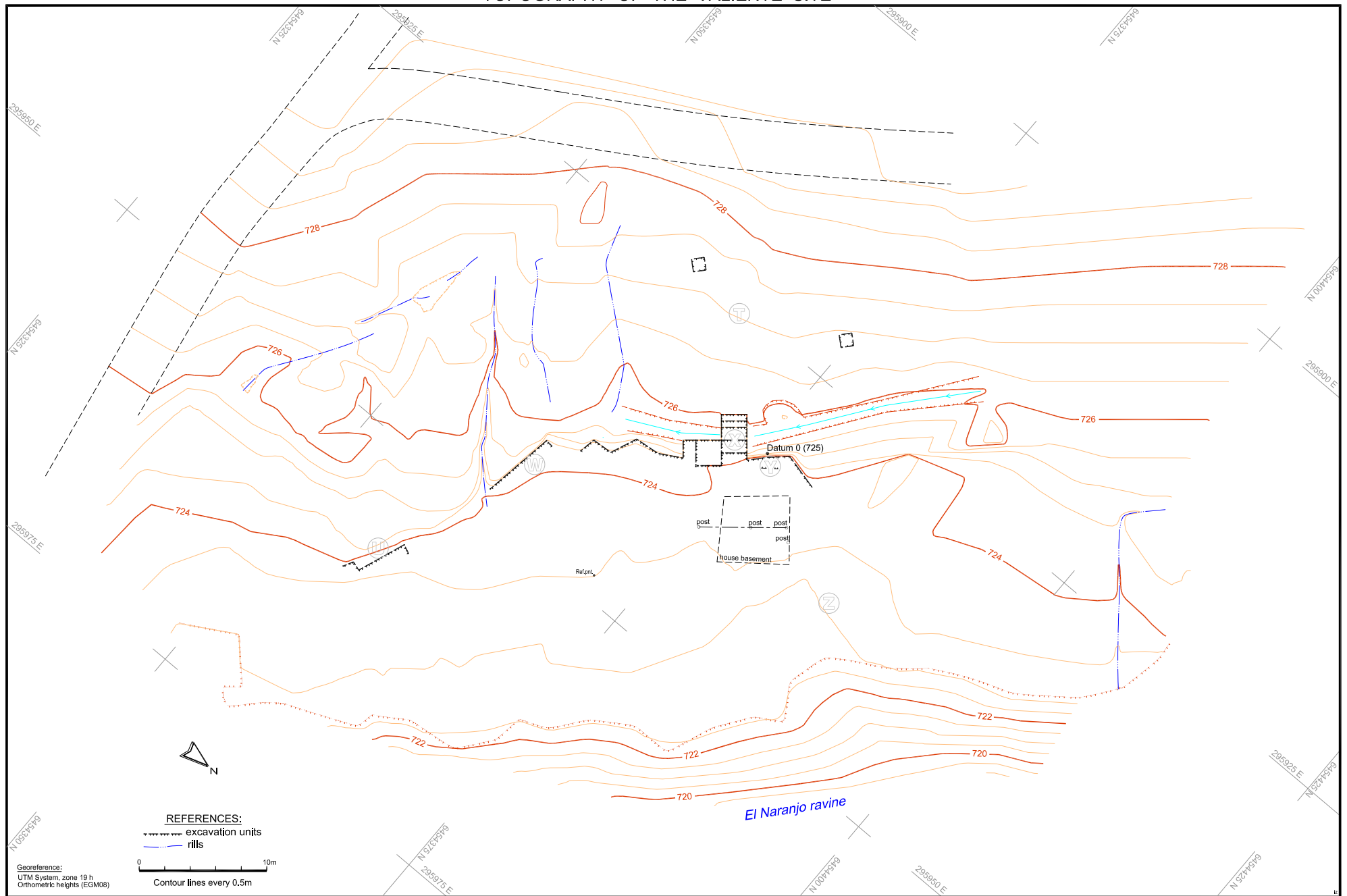

Supplement: S1 Fig — (PDF) [file pone.0208062.s001.pdf]

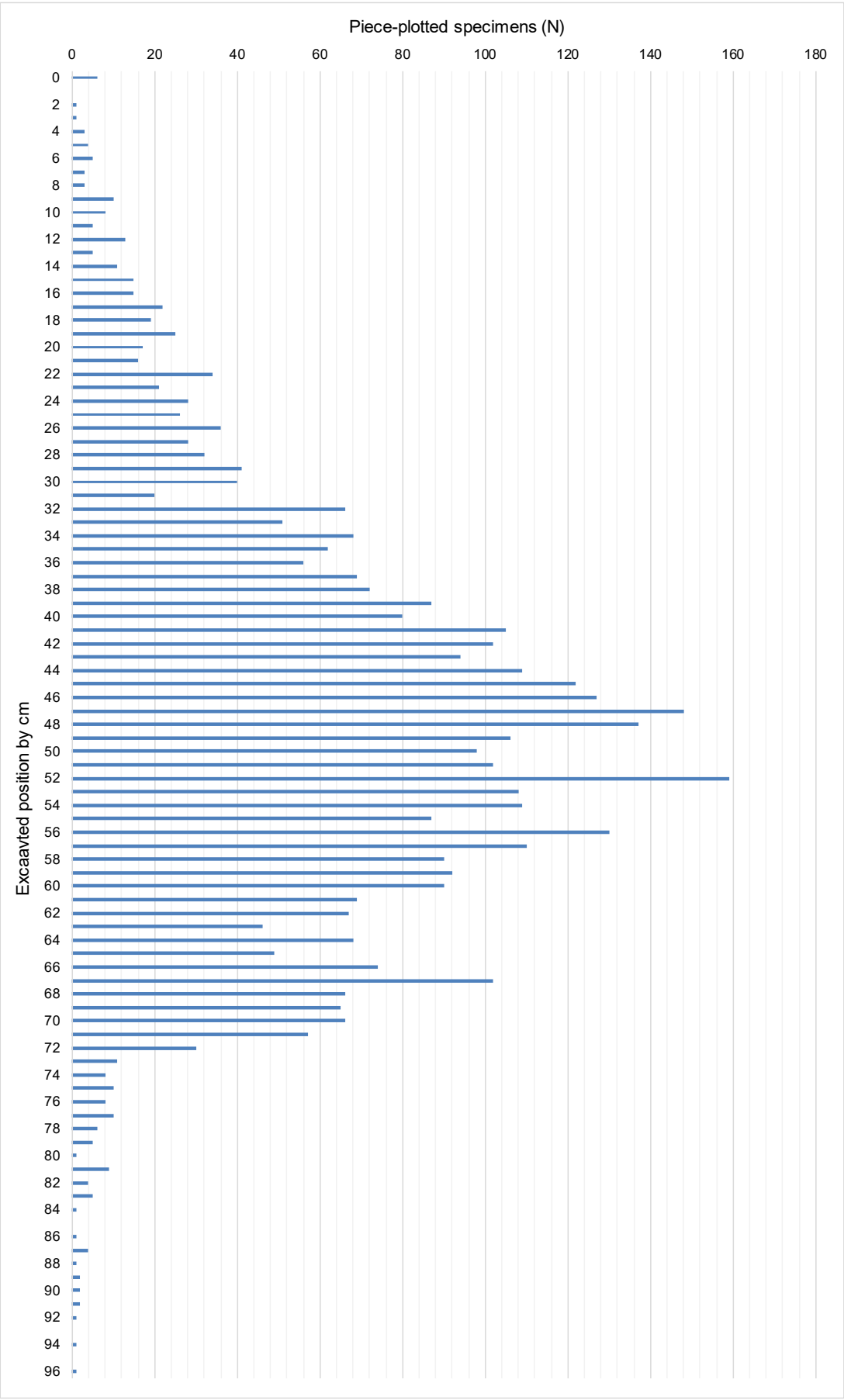

Supplement: S2 Fig — (PDF) [file pone.0208062.s002.pdf]

# Lithic distributions per excavated unit of area X partitioned by level

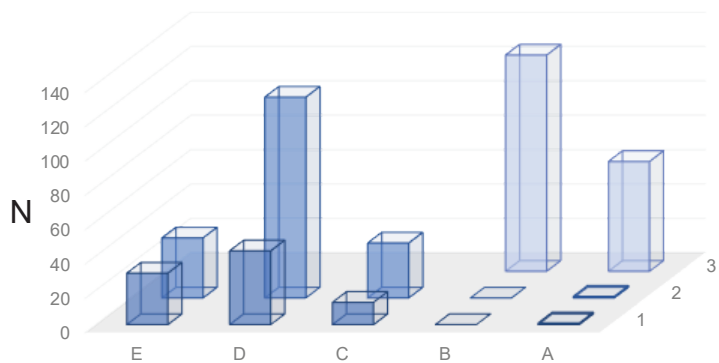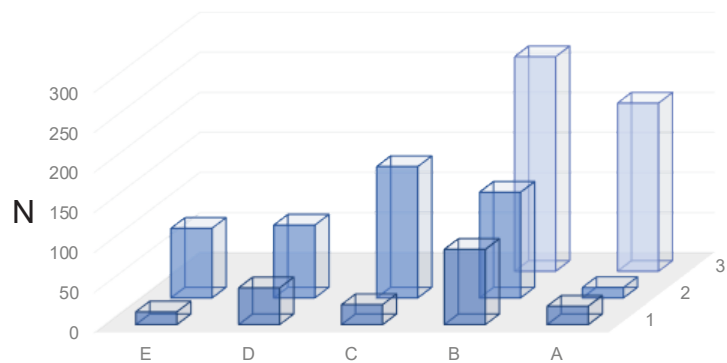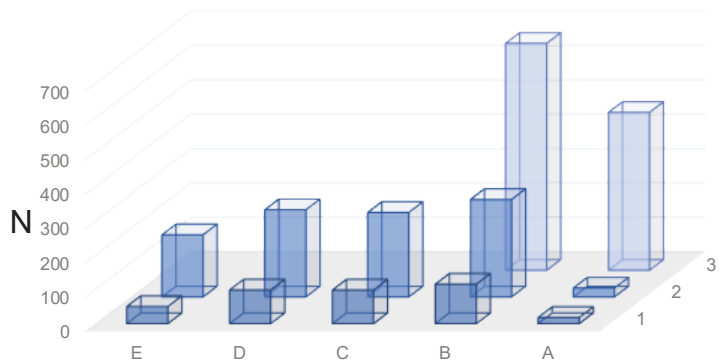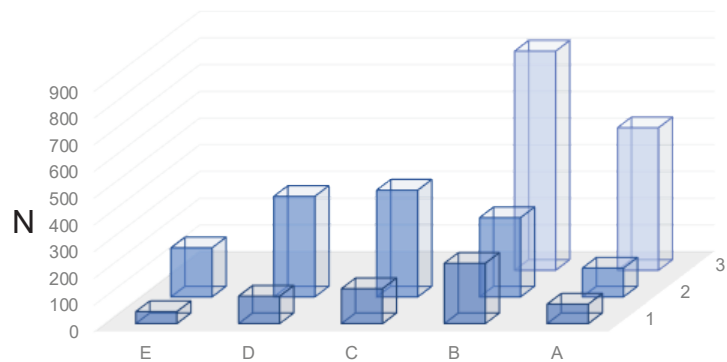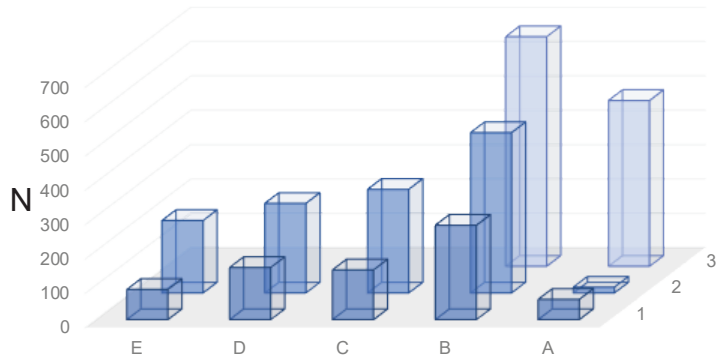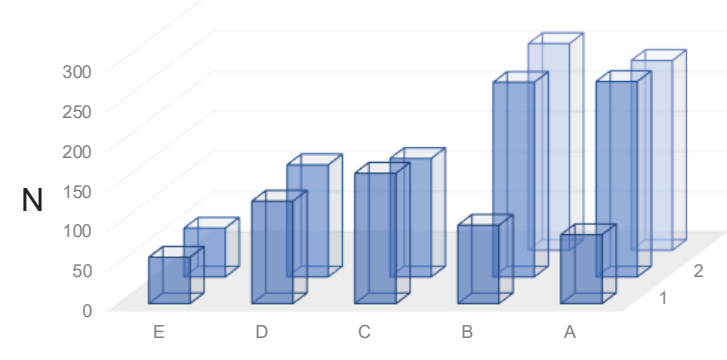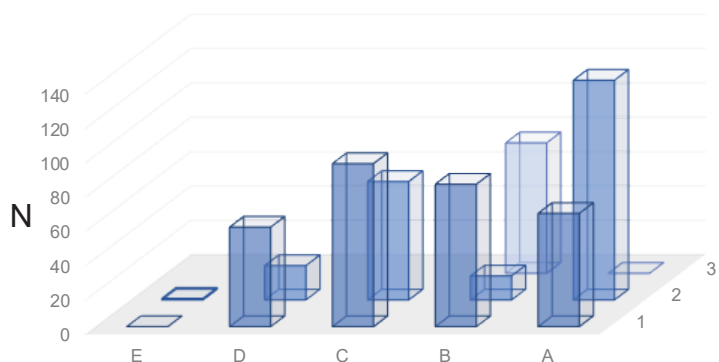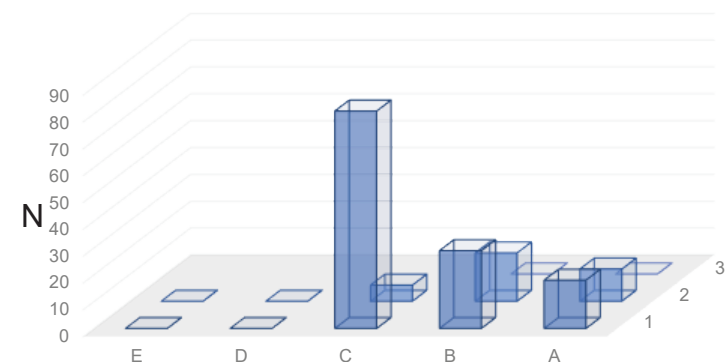

Supplement: S3 Fig — (PDF) [file pone.0208062.s003.pdf]

3D reconstruction of piece-plotted specimens from area X

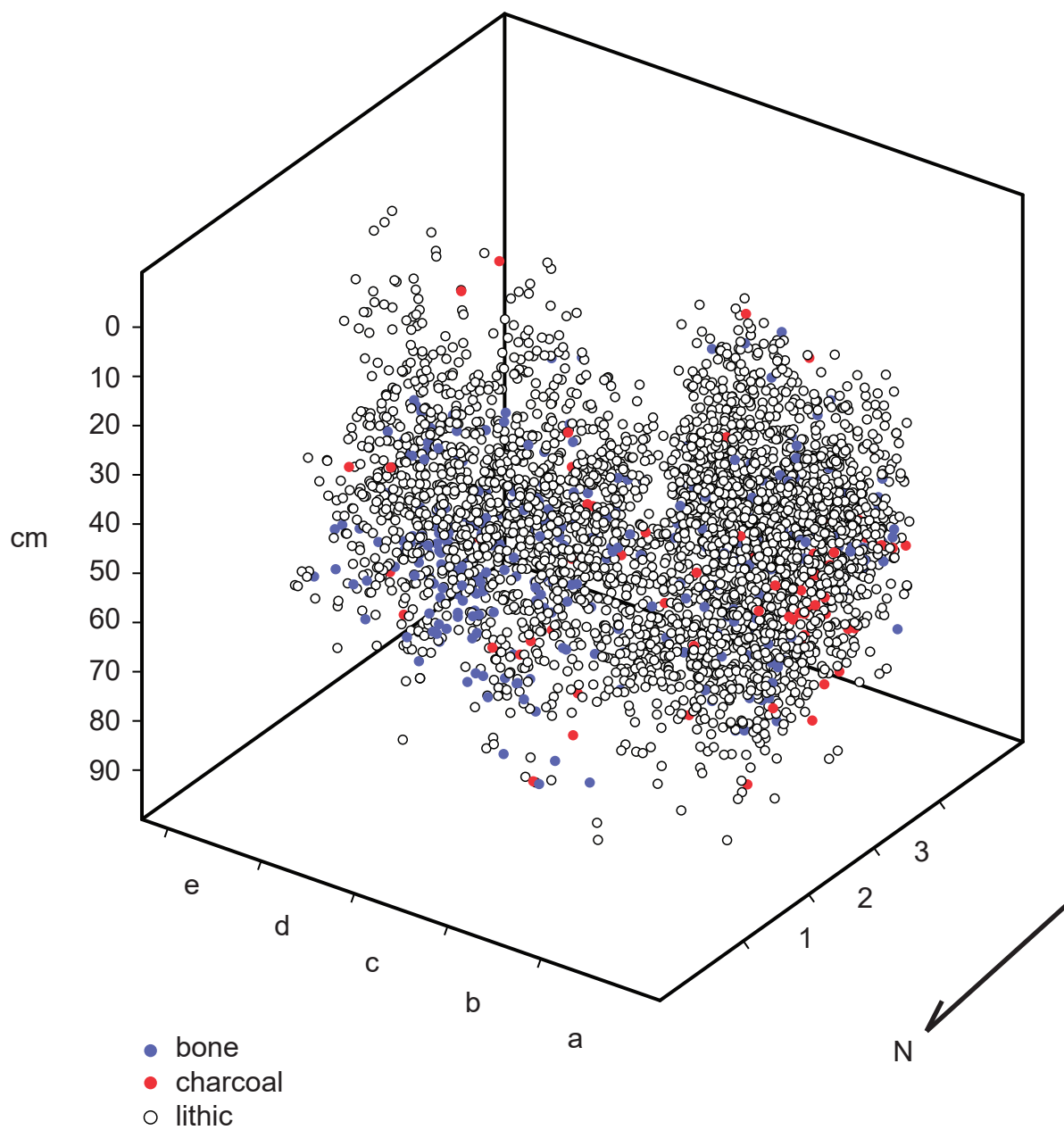

Supplement: S4 Fig — Vertical (depth) axis is exaggerated. (PDF) [file pone.0208062.s004.pdf]

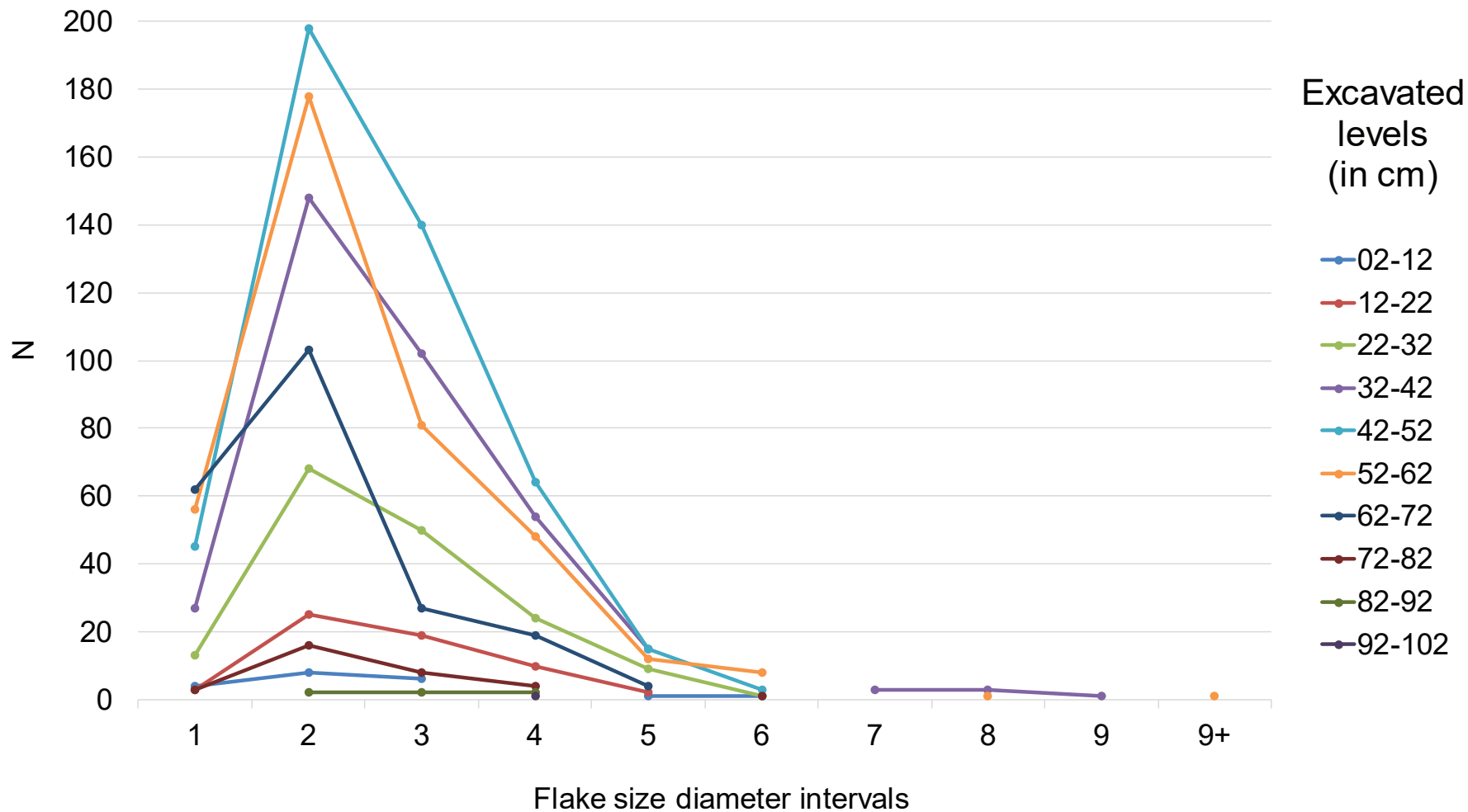

Supplement: S5 Fig — Intervals in X axis represent 1 cm increase. (PDF) [file pone.0208062.s005.pdf]

A.

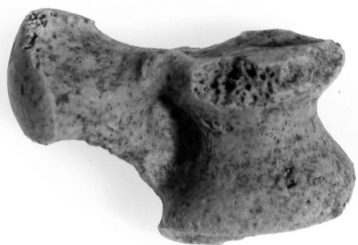

B.

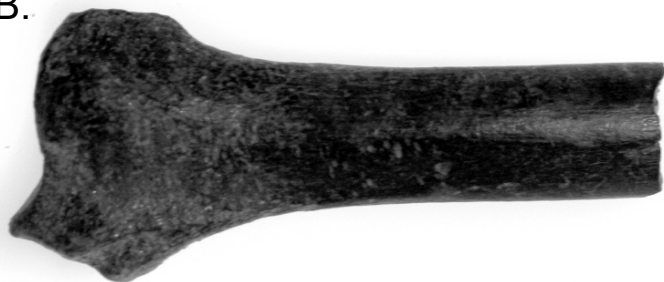

C.

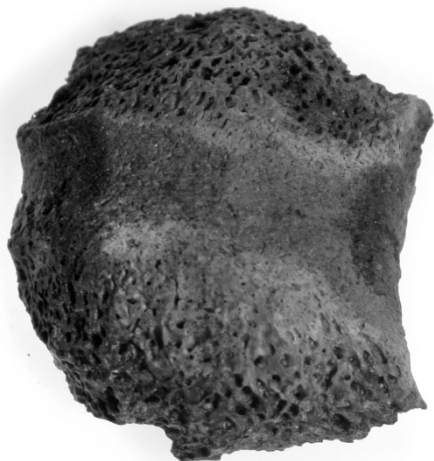

D.

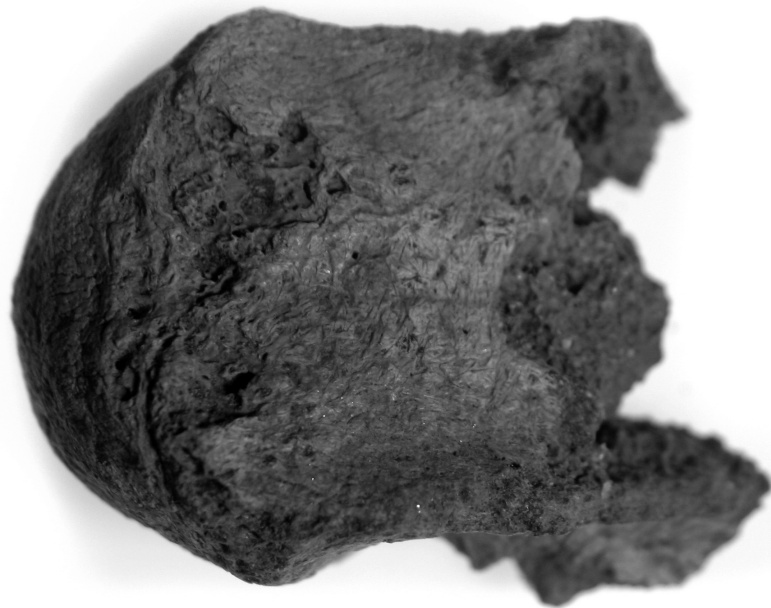

E.

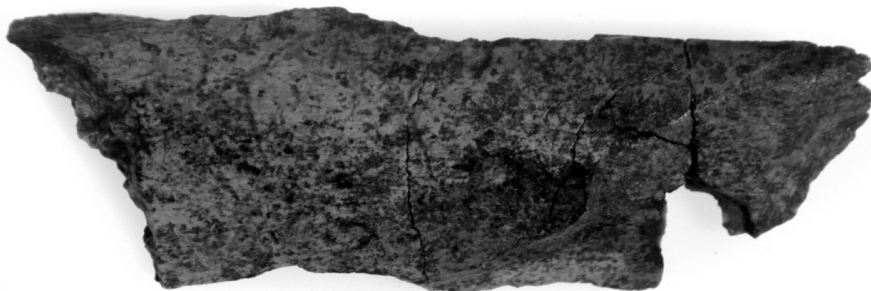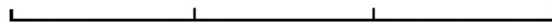

3 cm

Supplement: S6 Fig — A. left astragalus, Lycalopex griseus; B. radius, Lycalopex griseus; C. distal end of femur, Lycalopex griseus; D. vertebra fragment, Artiodactyla; E. indeterminate long bone fragment, Mammalia. (PDF) [file pone.0208062.s006.pdf]
