# Supplementary material for: Late Pleistocene to early Holocene high-quality quartz crystal procurement from the Valiente quarry workshop site (32°S, Chile, South America)
Source: PLoS One. 2018 Nov 29;13(11):e0208062. doi: 10.1371/journal.pone.0208062 (PMC6264839; doi:10.1371/journal.pone.0208062)
Supplement: S1 Appendix — (PDF) [file pone.0208062.s007.pdf]

Detailed stratigraphy of Area X at the Valiente site.

The stratigraphic section represented in excavation sector X is constrained to stratigraphic unit (SU) B. Five subunits were identified based on grain size, clast form and proportion, and color of the soil horizons (Fig 1).

SU B1 is constituted by quartz and feldspar sands, silts and clays with matrix supported subangular gravel of varied sizes. The larger fragments range between 0.5 and 3 cm (30% approx.). They are predominantly angular quartz fragments, with the occasional occurrence of rounded fragments of andesitic composition (3%). It is the oldest unit in this profile and covers a remnant pediment located between the slope and the alluvial terrace. It bears the main anthropogenic deposit.

SU B2 is constituted by quartz and feldspar sand, silt and clay with matrix supported subangular gravel of varied sizes. The larger fragments range from 0.5 to 30 cm as shown during the excavation of the first three artificial levels of the eastern sector of area X (excavation units D1, D2, E1, E2; Fig 2). These are predominantly angular quartz fragments. This subunit corresponds to an erosive rill in SU B1 and the subsequent filling of the cavity with alluvial deposits. Lesser quantity of lithic artifacts was observed in this unit during excavation.

SU B3 corresponds to a type-A soil horizon as indicated by a darker color and the presence of organic matter in the top section showing a gradual change to the base. The UGAMS 7819 date obtained at a 10 cm depth in SU B1, close to the contact with this horizon indicates its relatively recent formation (280-10 cal BP, Table 1).

SU B4 corresponds to an anthropogenic channel filled with clay, silt, sand, and gravel. Localized pedogenetic processes occur along its contour. It was probably excavated as a safety complement to the collapsed shack north of the excavation (Fig 2).

SU B5 are clay, silt, sand and gravels that correspond to recent hillside deposits of anthropic origin located only in the westernmost sector, close to area Y.

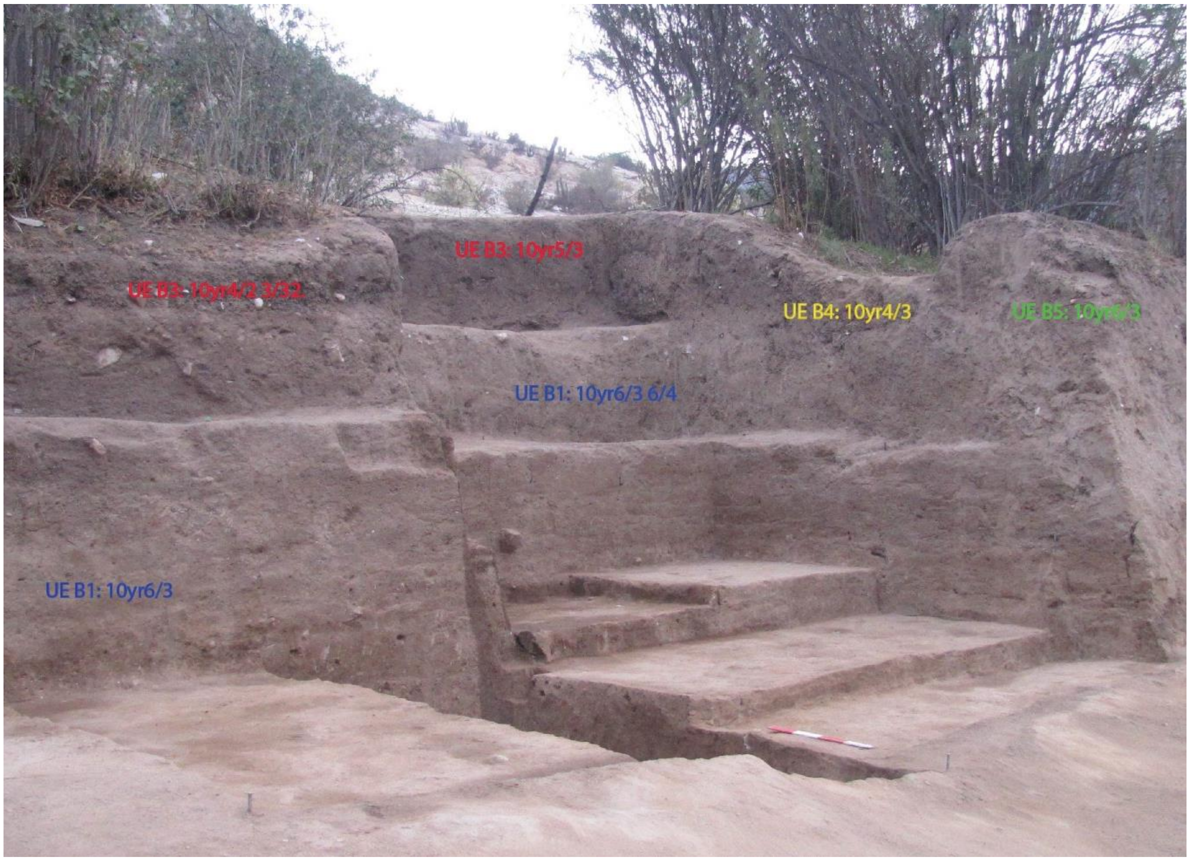

Fig 1. Excavation of Area X at the Valiente site. Munsell chart colors are shown in the different stratigraphic subunits.

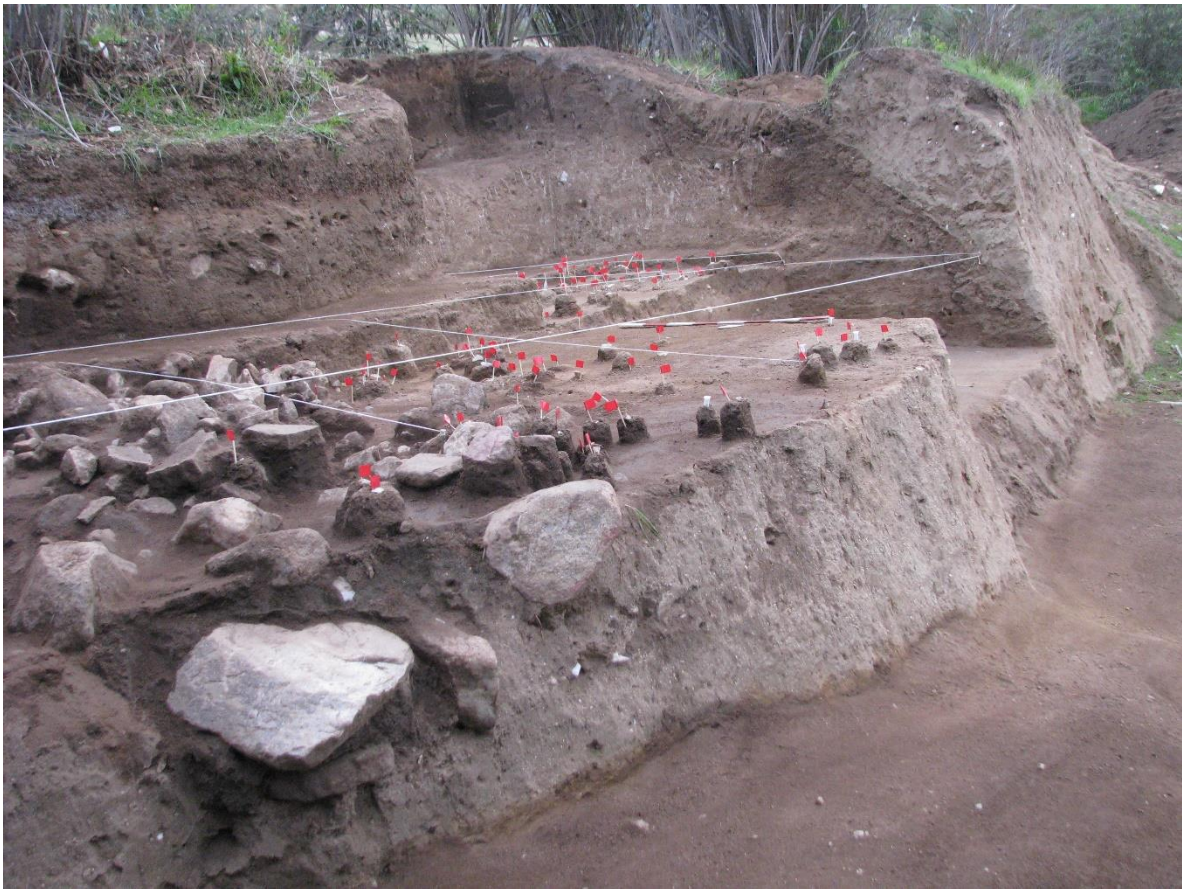

Fig 2. Detailed photograph of the filled erosive rill SU B2 as it was expressed in the excavation of the east sector of area X.
