# Supplementary material for: Late Pleistocene to early Holocene high-quality quartz crystal procurement from the Valiente quarry workshop site (32°S, Chile, South America)
Source: PLoS One. 2018 Nov 29;13(11):e0208062. doi: 10.1371/journal.pone.0208062 (PMC6264839; doi:10.1371/journal.pone.0208062)
Supplement: S2 Appendix — (PDF) [file pone.0208062.s008.pdf]

The bioarchaeological context of Area U at the Valiente site.

The excavation of stratigraphic unit B in area U produced a highly fragmented partial human skeleton whose remains indicate some were still in anatomical position by the time they were recorded (Fig 1). The remains were partially covered by sediments which clogged the medullar cavity of long bones. Osteological material was treated with standard conservation procedures and minimally restored for key measurements. Macroscopic analyses considered age estimations using teeth wear patterns (Lovejoy 1985), diagnoses of bone pathologies (Ortner 2003), and lifeway indicators (Aspillaga et al. 2006).

The human bone assemblage included small fragments of the temporal and frontal regions and fragments of cervical vertebrae. Only remains of 8 teeth were recovered from the excavated unit, despite the occurrence of several fragments in the exposed profile north of the excavated unit (Fig 2). More than eighty individual fragments of between 2 mm^2 to 1 cm^2 comprise a group of highly fragmented hand bones. Several bone fragments of the right foot were also recovered. Among the better represented skeletal remains are the two tibia and the two femurs. However, these were only represented by the diaphysis sections as expected with less segments of less bone mineral density (epiphysis).

Despite the high fragmentation and the occurrence of a limited bone assemblage, it can be concluded that the remains belong to a single individual. Their incomplete state is mainly due to postdepositional disturbances, chiefly the placing of a huge (>50 cm) stone on top of the burial. All expected remains beneath it were completely absent.

Only limited information was obtained. Tooth wear is consistent with C and D stages which roughly correspond to an age between 20 and 24 years (Lovejoy 1985). Wear can be associated with a rich fiber diet causing chipping of the enamel, a sign of a diet with high protein intake. Possible parafunctional wear was observed only in one tooth. Relative diaphysis sizes and gracile bone attributes are consistent with the individual as an adult female, although this should be regarded as preliminary. No evident pathologies were observed, except for possible osteitis in one hand bone which suggests functional stress.

A direct date on an upper first molar fragment produced an age of 5930-5740 cal BP (see main text). Stable isotope analyses on the same sample yielded values of $\delta^{13}C_{ap}$: -9.9‰, $\delta^{15}N$: 8.7‰, and $\delta^{13}C_{coll}$: -17.7 ‰ (UGAMS 7818). When compared to other isotopic results on local skeletal material and on selected resources from the broader region, this individual shows diets consistent with inland protein intake expected for a hunter gatherer economy (Falabella et al. 2007; Jackson et al. 2012; Gómez and Pacheco 2016).

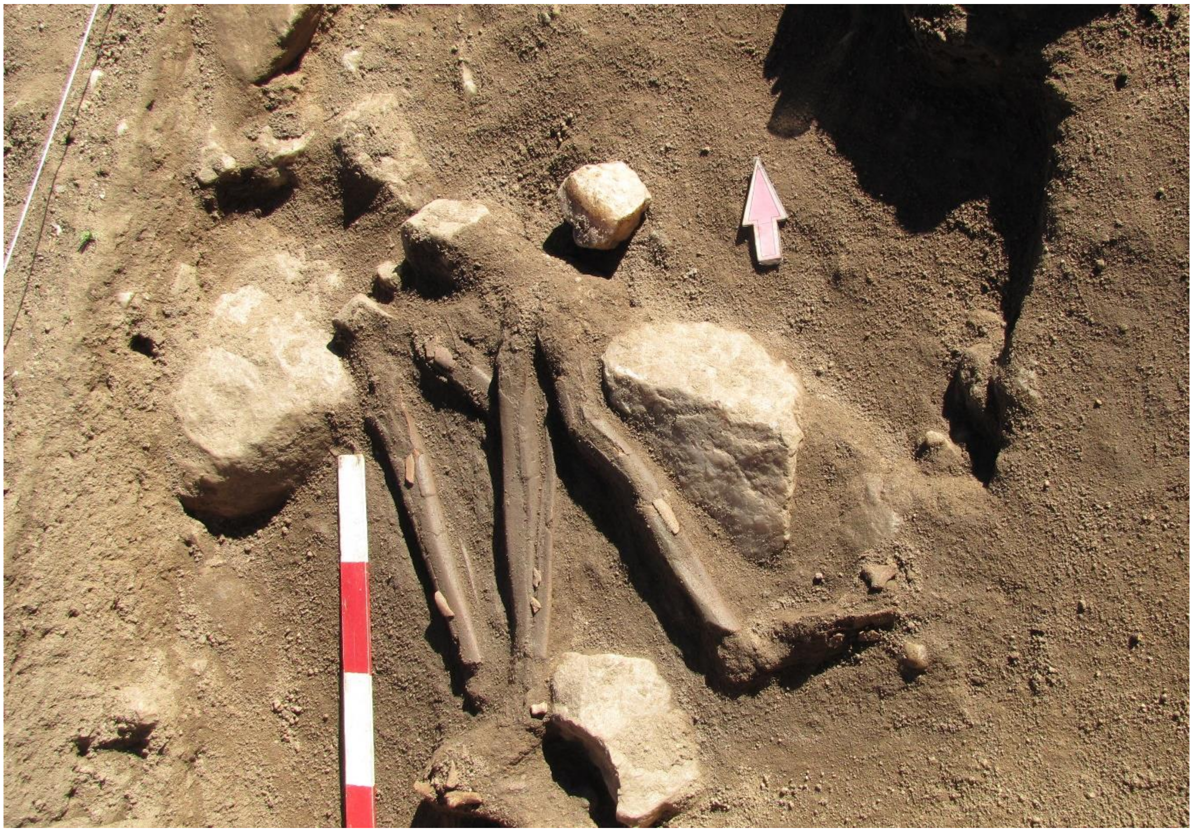

Fig 1. Photograph of the human remains excavated feature at area U.

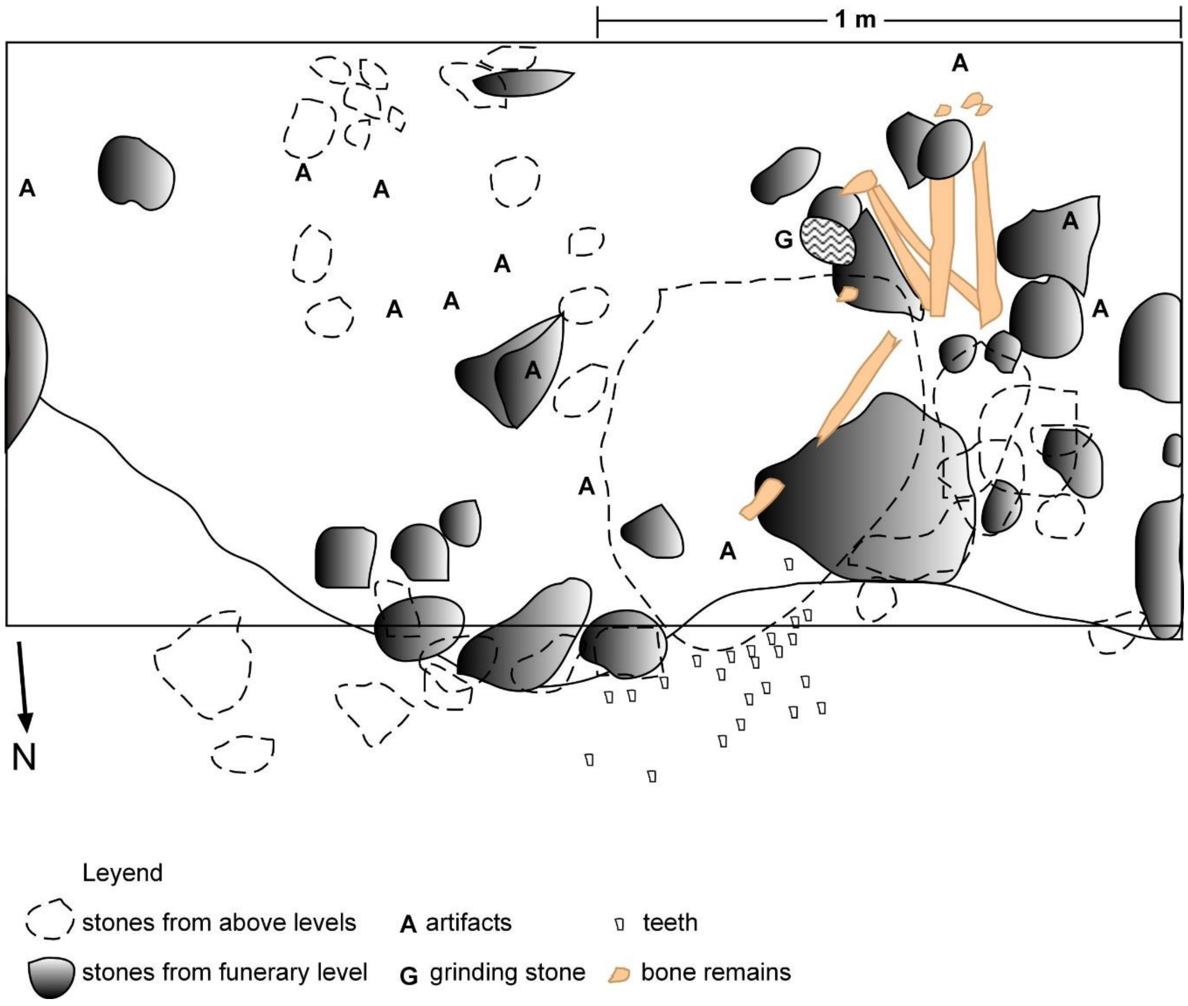

Fig 2. Excavation plan of area U depicting the associated context.

References

Aspillaga E, Castro M, Rodríguez M, Ocampo C. Paleopatología y estilo de vida: El ejemplo de los Chonos. Magallania 2006; 34: 77-85.

Falabella F, Planella MT, Aspillaga E, Sanhueza L, Tykot R. Dieta en sociedades alfareras de Chile Central: Aporte de análisis de isótopos estables. Chungara Revista de Antropología Chilena 2007; 39: 5-27.

Gómez P, Pacheco A. Movilidad y dieta en el Valle de El Mauro (31°57'S. – 71°01'W.), Norte semiárido de Chile, entre 8350–929 CAL. AP. Comechingonia 2016; 20: 51-79.

Jackson D, Méndez C, Aspillaga E. Human remains directly dated to the Pleistocene-Holocene transition support a maritime diet among the first settlers of the Pacific coast of South America. Journal of Island and Coastal Archaeology 2012; 7: 363-377.

Lovejoy OC. Dental wear in the Libben population: Its functional pattern and role in the determination of adult skeletal age at death. American Journal of Physical Anthropology 1985; 68: 47-56.

Ortner D. Identification of pathological conditions in human skeletal remains. 2nd ed. San Diego, CA.: Academic Press; 2003.
