# Supplementary material for: Late Pleistocene to early Holocene high-quality quartz crystal procurement from the Valiente quarry workshop site (32°S, Chile, South America)
Source: PLoS One. 2018 Nov 29;13(11):e0208062. doi: 10.1371/journal.pone.0208062 (PMC6264839; doi:10.1371/journal.pone.0208062)
Supplement: S3 Appendix — (PDF) [file pone.0208062.s009.pdf]

# Scanning Electron Microscopy of faunal material of Area X at the Valiente site.

Bone samples were selected form the excavation units with best chronological control and from the levels that produced most bone specimens. All samples were visibly black. Results from the SEM-EDS analysis are shown in table 1 and figures 1 through 6. All the samples gave a positive spectrum for the presence of iron and manganese, except the sample form unit B2/level 42-52 cm, which only yielded iron (Fig 3).

Manganese precipitation occurs when there is saturation of this element in water associated with a lack of oxygen (Martin 2013). We interpret that the compounds correspond to manganese dioxide ($MnO^2$) and ferric oxide. Manganese dioxide and ferrous oxide, most likely ferric ferrous oxide, blackened the bones on the surface and in the medullary cavity, infiltering through the haversian canals and interstices, but did not affect the compact bone. Therefore, some allegedly burnt bone fragments may be masked by the black staining of the manganese dioxide and ferric oxide.

## References

Martin FM. Tafonomía de la Transición Pleistoceno-Holoceno en Fuego-Patagonia. Interacción entre Humanos y Carnívoros y su Importancia como Agentes en la Formación del Registro Fósil. Punta Arenas: Universidad de Magallanes; 2013. 406 p.

**Table 1. Chemical composition (mass percent %) of bone samples of Area X at the Valiente site.**

| Sample Unit/level | Analysis | Chemical elements | | | | | | | | | | | |
|---|---|---|---|---|---|---|---|---|---|---|---|---|---|
| | | O | F | Na | Mg | Al | Si | P | K | Ca | Ti | Mn | Fe |
| B1/22-32 cm | 1 | 43.89 | - | 0.15 | 1.89 | - | 14.29 | 3.31 | 2.29 | 13.79 | 2.25 | 1.64 | 16.5 |
| | 2 | 39.34 | - | 0.56 | 1.51 | 13.08 | 20.13 | - | 1.78 | 7.71 | 0.39 | 0.88 | 14.61 |
| | 3 | 43.54 | - | 0.34 | 0.2 | - | 0.87 | 14.62 | - | 37.63 | | 1.29 | 1.52 |
| | 4 | 44.2 | - | 0.33 | 2.23 | - | 21.29 | - | 4.36 | 8.7 | 1.46 | 0.35 | 17.08 |
| | 5 | 40.94 | - | | 6.24 | 13.91 | 19.8 | - | 1.05 | 0.4 | | 0.33 | 17.34 |
| | Mean | 42.38 | - | 0.35 | 2.41 | 13.49 | 15.28 | 8.97 | 2.37 | 13.64 | 1.37 | 0.9 | 13.41 |
| | Sigma | 2.14 | - | 0.17 | 2.27 | 0.58 | 8.5 | 8 | 1.42 | 14.23 | 0.93 | 0.58 | 6.73 |
| | Sigma mean | 0.96 | - | 0.07 | 1.02 | 0.26 | 3.8 | 3.58 | 0.64 | 6.37 | 0.42 | 0.26 | 3.01 |
| B1/32-42 cm | 1 | 35.67 | - | 0.27 | 0.23 | - | 0.25 | 15.1 | - | 46.18 | - | 1.45 | 0.85 |
| | 2 | 48.94 | - | 0.49 | 0.19 | - | - | 13.47 | - | 35.78 | - | 1.13 | |
| | 3 | 47.71 | - | 0.74 | 0.51 | - | 3.5 | 12.28 | 0.17 | 30.32 | - | 2.3 | 2.46 |
| | Mean | 44.11 | - | 0.5 | 0.31 | - | 1.88 | 13.62 | 0.17 | 37.43 | - | 1.63 | 1.66 |
| | Sigma | 7.33 | - | 0.24 | 0.17 | - | 2.3 | 1.42 | 0 | 8.05 | - | 0.61 | 1.14 |
| | Sigma mean | 4.23 | - | 0.14 | 0.1 | - | 1.33 | 0.82 | 0 | 4.65 | - | 0.35 | 0.66 |
| B2/42-52 cm | 1 | 50.2 | - | 0.66 | 0.58 | - | 1.12 | 13.48 | - | 33.43 | - | | 0.53 |
| | 2 | 32.83 | - | | 0.24 | - | 1.04 | 16.15 | - | 49.07 | - | | 0.67 |
| | 3 | 44.43 | - | 0.42 | 0.54 | - | 0.66 | 15.56 | - | 37.96 | - | | 0.42 |
| | Mean | 42.49 | - | 0.54 | 0.45 | - | 0.94 | 15.06 | - | 40.15 | - | | 0.54 |
| | Sigma | 8.85 | - | 0.17 | 0.18 | - | 0.25 | 1.4 | - | 8.04 | - | | 0.12 |
| | Sigma mean | 5.11 | - | 0.1 | 0.11 | - | 0.14 | 0.81 | - | 4.64 | - | | 0.07 |

| Sample Unit/level | Analysis | Chemical elements | | | | | | | | | | | |
|---|---|---|---|---|---|---|---|---|---|---|---|---|---|
| | | O | F | Na | Mg | Al | Si | P | K | Ca | Ti | Mn | Fe |
| B1/52-62 cm | 1 | 48.79 | 0.14 | 0.81 | 0.4 | - | - | 15.01 | - | 34.48 | - | 0.21 | 0.16 |
| | 2 | 43.86 | 3.4 | 0.62 | 0.32 | 0.14 | - | 15.44 | - | 36.23 | - | | |
| | 3 | 46.37 | 1.72 | 0.66 | 0.46 | 0.65 | 0.6 | 14.89 | - | 34.07 | - | | 0.57 |
| | Mean | 46.34 | 1.76 | 0.7 | 0.39 | 0.4 | 0.6 | 15.11 | - | 34.93 | - | 0.21 | 0.36 |
| | Sigma | 2.47 | 1.63 | 0.1 | 0.07 | 0.36 | 0 | 0.29 | | 1.15 | | 0 | 0.29 |
| | Sigma mean | 1.43 | 0.94 | 0.06 | 0.04 | 0.21 | 0 | 0.17 | | 0.66 | | 0 | 0.17 |
| A1/62-72 cm | 1 | 40.43 | | 0.37 | 0.16 | 0.14 | | 16.05 | | 42.46 | | 0.38 | |
| | 2 | 38.36 | | 0.19 | 0.08 | | 1.72 | 14.27 | | 41.55 | | 1.04 | 2.28 |
| | 3 | 42.27 | | | 0.09 | | 2.1 | 13.95 | | 39.94 | | | 1.65 |
| | Mean | 40.52 | | 0.28 | 0.11 | 0.14 | 1.91 | 14.76 | | 41.32 | | 0.71 | 1.97 |
| | Sigma | 1.7 | | 0.13 | 0.04 | 0 | 0.27 | 1.13 | | 1.28 | | 0.46 | 0.45 |
| | Sigma mean | 0.98 | | 0.07 | 0.03 | 0 | 0.16 | 0.65 | | 0.74 | | 0.27 | 0.26 |
| A2/72-82 cm | 1 | 51.84 | | 1.49 | 0.81 | | 11.93 | 5.56 | 0.76 | 18.4 | 0.14 | 0.21 | 8.87 |
| | 2 | 49.19 | | 0.53 | 0.6 | | 8.45 | 6.67 | 0.52 | 19.62 | | 3.21 | 11.22 |
| | 3 | 46.11 | | 0.4 | 0.24 | | 0.48 | 14.81 | | 37.63 | | | 0.34 |
| | 4 | 47.3 | | 0.35 | 0.25 | | 1.06 | 13.64 | | 36.63 | | | 0.77 |
| | Mean | 48.61 | | 0.69 | 0.47 | | 5.48 | 10.17 | 0.64 | 28.07 | 0.14 | 1.71 | 5.3 |
| | Sigma | 2.5 | | 0.54 | 0.28 | | 5.63 | 4.73 | 0.17 | 10.48 | 0 | 2.12 | 5.57 |
| | Sigma mean | 1.25 | | 0.27 | 0.14 | | 2.81 | 2.36 | 0.08 | 5.24 | 0 | 1.06 | 2.78 |

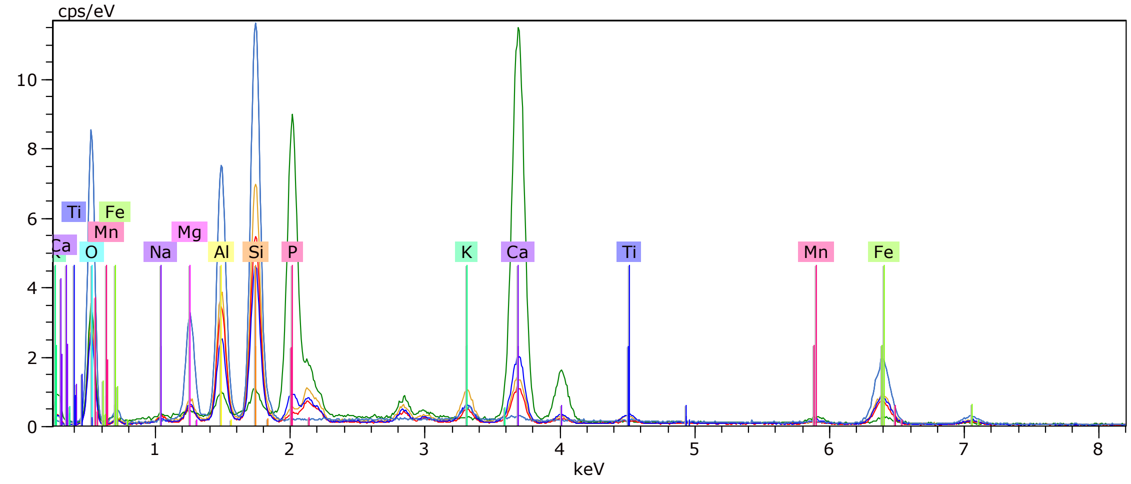


**Fig 1. Bone sample unit B1, 22-32 cm.**

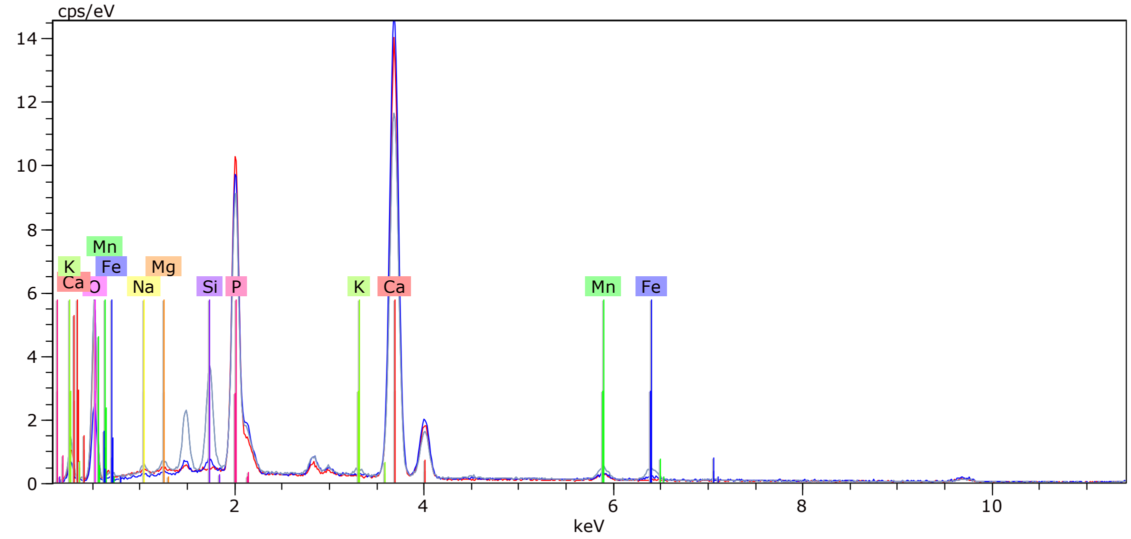


**Fig 2. Bone sample unit B1, 32-42 cm.**

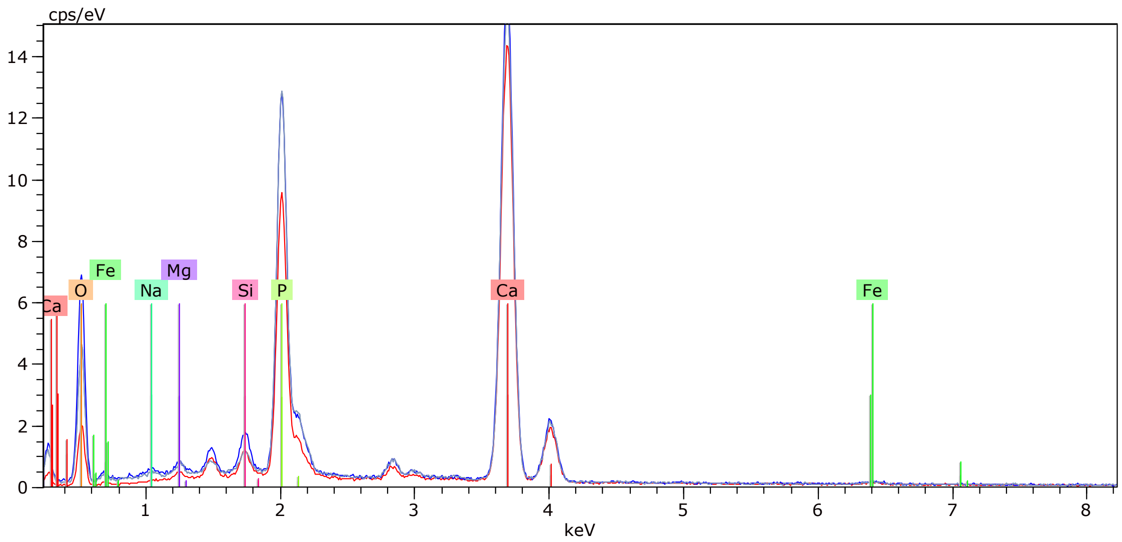


**Fig 3. Bone sample unit B2, 42-52 cm.**

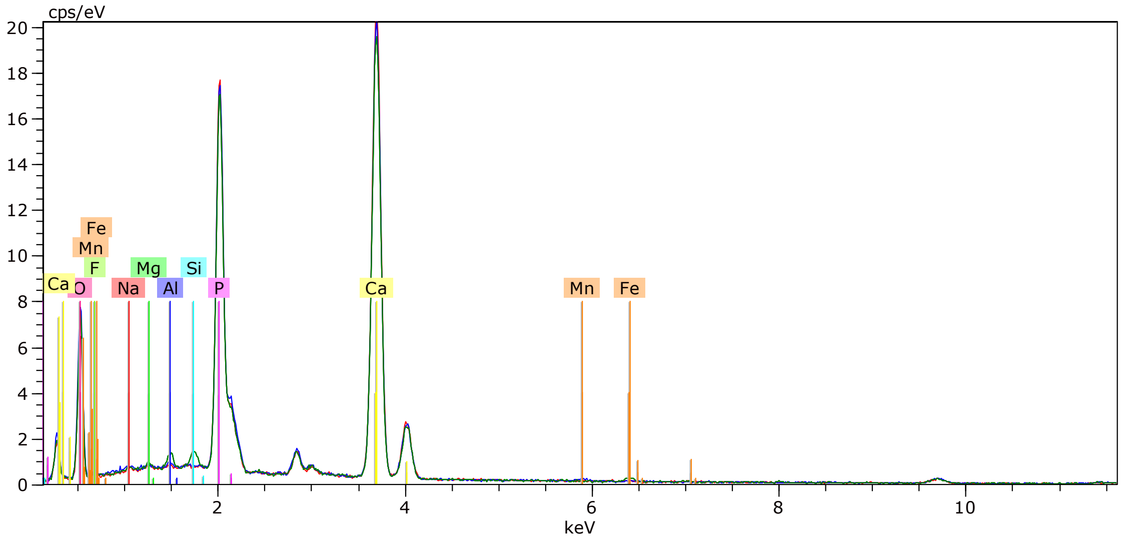


**Fig 4. Bone sample unit B1, 52-62 cm.**

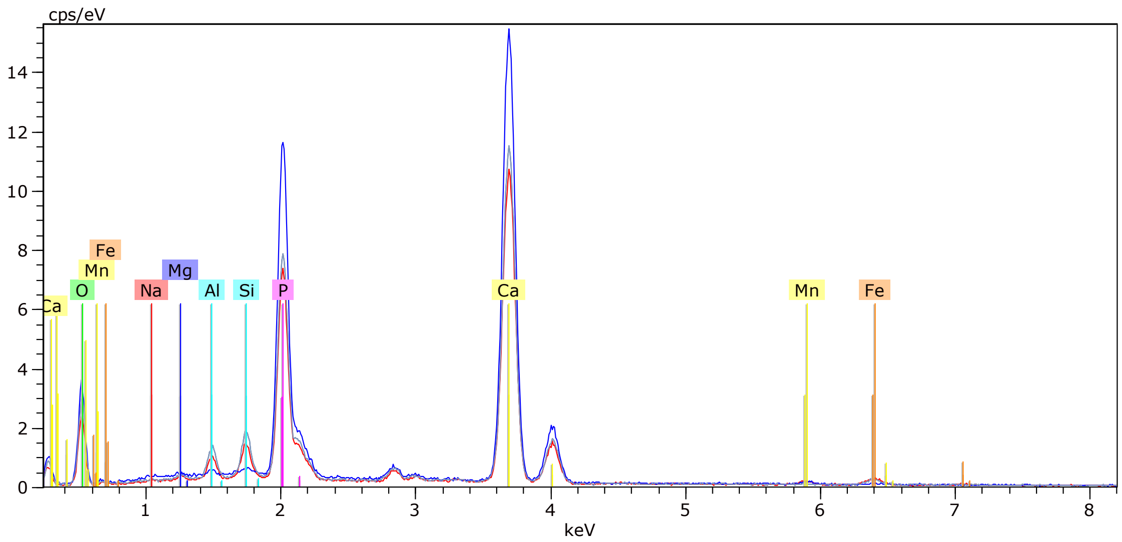


**Fig 5. Bone sample unit A1, 62-72 cm.**

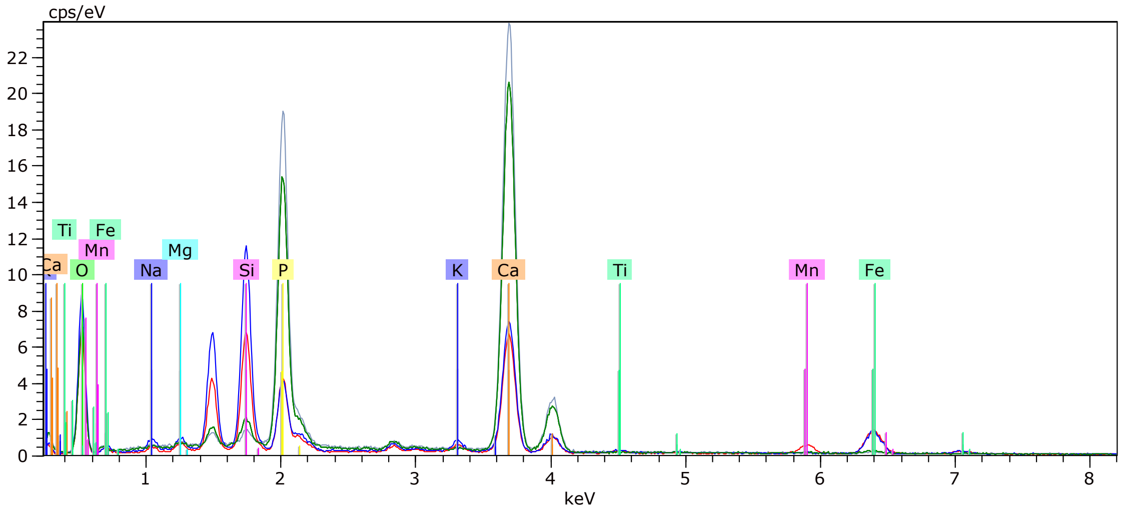


**Fig 6. Bone sample unit A2, 72-82 cm.**
